# Identifying the potential of anadromous salmonid habitat restoration with life cycle models

**DOI:** 10.1371/journal.pone.0256792

**Published:** 2021-09-09

**Authors:** Jeffrey C. Jorgensen, Colin Nicol, Caleb Fogel, Timothy J. Beechie

**Affiliations:** 1 Fish Ecology Division, Northwest Fisheries Science Center, National Marine Fisheries Service, National Oceanic and Atmospheric Administration, Seattle, Washington, United States of America; 2 Ocean Associates, Inc., Under contract to Northwest Fisheries Science Center, National Marine Fisheries Service, National Oceanic and Atmospheric Administration, Seattle, Washington, United States of America; University of Waikato, NEW ZEALAND

## Abstract

An investigation into the causes of species decline should include examination of habitats important for multiple life stages. Integrating habitat impacts across life stages with life-cycle models (LCMs) can reveal habitat impairments inhibiting recovery and help guide restoration efforts. As part of the final elements of the Habitat Restoration Planning model (HARP; Beechie et al. this volume), we developed LCMs for four populations of three species of anadromous salmonids (*Oncorhynchus kisutch*, *O*. *tshawytscha*, and *O*. *mykiss*), and ran diagnostic scenarios to examine effects of barrier removal, fine sediment reduction, wood augmentation, riparian shade, restoration of the main channel and bank conditions, beaver pond restoration, and floodplain reconnection. In the wood scenario, spawner abundance for all populations increased moderately (29–48%). In the shade scenario, spring-run Chinook salmon abundance increased the most (48%) and fall-run Chinook salmon and steelhead were much less responsive. Coho responded strongly to the beaver pond and floodplain scenarios (76% and 54%, respectively). The fine sediment scenario most benefitted fall- and spring-run Chinook salmon (32–63%), whereas steelhead and coho were less responsive (11–21% increase). More observations are needed to understand high fine sediment and its impacts. Our LCMs were region-specific, identifying places where habitat actions had the highest potential effects. For example, the increase in spring-run Chinook salmon in the wood scenario was driven by the Cascade Mountains Ecological Region. And, although the overall response of coho salmon was small in the barrier removal scenario (6% increase at the scale of the entire basin), barrier removals had important sub-regional impacts. The HARP analysis revealed basin-wide and regional population-specific potential benefits by action types, and this habitat-based approach could be used to develop restoration strategies and guide population rebuilding. An important next step will be to ground-truth our findings with robust empirically-based estimates of life stage-specific survivals and abundances.

## Introduction

Efforts to conserve imperiled species begin with an investigation of potential causes of decline, including analyses of the effects of habitat loss or degradation on populations [1]. Key habitats include those used for reproduction, growth, and rearing, as well as for migration. After impaired habitats are identified, a process follows that establishes recommendations for habitat remediation:, including the kinds of actions to be taken, where actions can plausibly be implemented, and the extent and magnitude of actions needed to remove or substantially mitigate the identified impairments [2]. For freshwater aquatic species, a substantial amount of resources and efforts are often required to correct root causes of habitat degradation. Because of the high costs associated with implementing some potential actions, it is also important to prioritize actions and improve cost effectiveness of restoration efforts [2]. A prioritized set of actions comprises a habitat remediation strategy.

An important first step in establishing action prioritization is to develop an understanding of the links between habitat remediation actions and population-level responses for target species [3]. This can be accomplished by quantitatively linking detailed characterization of habitats used by animals at particular life stages with population viability or life cycle models (LCM) that use demographic parameters governing the entire lifespan and that reflect changes to habitat (e.g., [4–7]). Linking habitat change to life stage-specific parameters in a life cycle model enables prioritization of habitat remediation actions by examining the extent to which each action potentially contributes to the biological response of populations. This represents the final steps in the habitat-based salmon Habitat Restoration Planning model (HARP model; Beechie et al. this volume). Because it is habitat-based, HARP can be developed for in basins where fish data are limited; however, the analysis benefits from as many parameter estimates as possible informed by population-specific observations.

The initial stages of the HARP model described in Beechie et al. (this volume) estimate the restoration potential for anadromous Pacific salmonid (*Oncorhynchus* spp.) habitats, and this study adds to that analysis with population- and spatially-specific estimates of biological response to habitat change for spring-run and fall-run Chinook salmon (*O*. *tshawytscha*), coho salmon (*O*. *kisutch*), and winter-run steelhead (*O*. *mykiss*) in the Chehalis River Basin (Washington State, USA). The motivation for HARP is to illustrate a coupled habitat-LCM approach in support of restoration planning to help planners understand how habitat-forming processes contribute to salmon responses, and to develop a restoration strategy that promotes the greatest positive population outcome.

For this part of the HARP model, we begin by describing the LCMs and a suite of diagnostic habitat scenarios informed by the habitat change analysis of Beechie et al. (this volume). We then use these diagnostic scenarios in LCMs to identify important restoration actions and, because the LCMs include habitat information at the regional level, locations where each type of restoration is likely to be most beneficial.

## Methods

The assessment of salmon habitat and habitat-forming processes in Beechie et al. (this volume) produced several data sets describing how habitats in the Chehalis River basin currently differ from their natural potentials. In this paper, we translated those current and natural potential habitat areas and conditions into population- and life stage-specific productivity (survival) and capacity estimates, which we used to parameterize age-structured, stage-based life cycle models (e.g., [8]). For the habitat-forming processes and habitat attributes assessed in Beechie et al. (this volume), we individually set each habitat condition to its full natural potential to create diagnostic scenarios to identify which habitat changes most constrain salmonid population production, and which restoration actions might provide the most benefit for each population.

## Study basin

**Fig 1:**
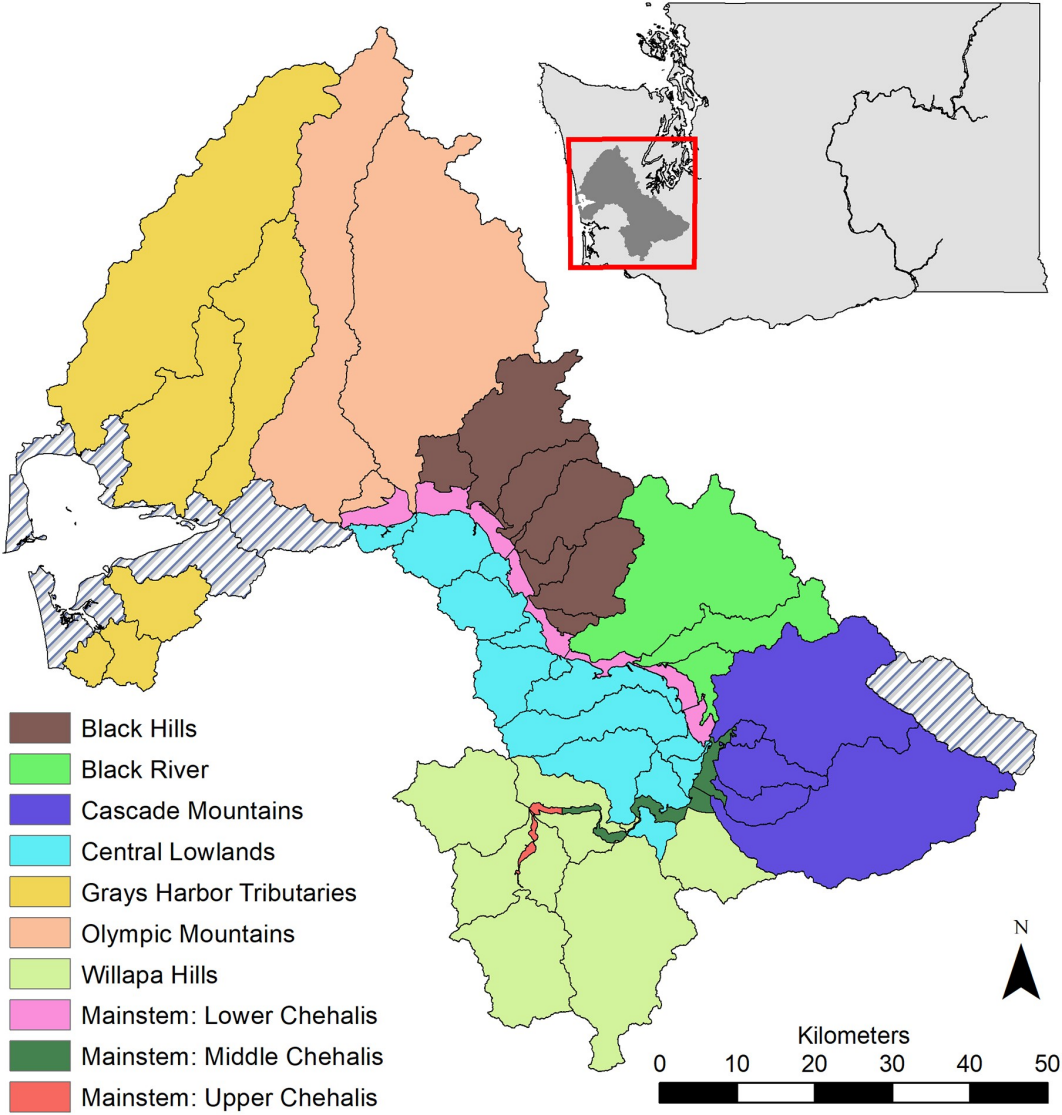
Map of the 63 subbasins (black boundaries) and Ecological Regions (colored polygons) as outlined in Beechie et al. (this volume). Subbasins representing tributaries to the mainstem Chehalis River were each modeled as individual subpopulations in the life cycle models, and Ecological Regions are unique geographic areas. Gray hatched subbasins were not included because they did not contain spawning salmon due to impassible barriers, or were part of the delta-bay (upper and lower estuary).

The Chehalis River Basin (southwestern Washington State, USA) drains an area of approximately 6,900 km^2^ (Fig 1). A more detailed description of the Chehalis River Basin can be found in Beechie et al. (this volume). For this analysis, we divided the basin into 63 distinct spatial units or subbasins, which are comprised of all the individual tributaries to the Chehalis River and to Grays Harbor, and included several mainstem Chehalis River spatial units. Mainstem units were delineated by the confluences of major tributaries, and which allowed us to account for movement of fish into and through the mainstem from the tributaries. All of these subbasins were assigned by the Chehalis Basin Scientific Review Team to 10 unique Ecological Regions according to similarities of subbasin geology and ecological characteristics.

## Life cycle model structure

The life cycle models are age-structured, stage-based population simulation models (e.g., (8)). Cohorts are tracked in both space and time, and by age class and location via an abundance array. The row dimension of the array corresponds to life-stage capacities and productivities, and the number of rows varies by population depending on population-specific life history and age structure (S1 in S1 File). The column dimension corresponds to the 63 freshwater spatial units into which the Chehalis basin is divided (subbasin or mainstem segment; Fig 1) (e.g., [9]). Juvenile production in each simulation year in each spatial unit is generated from spawners that return to that spatial unit.

As fish transition across life stages, they may remain in the same subbasin or move to a new location by migrating downstream to the mainstem, delta-bay (the estuary), or ocean. In some cases, movement was modeled as volitional or density-independent, and in other cases we used a density-dependent movement function (e.g., [10]). When fish moved downstream to the mainstem from a tributary subbasin, they joined downstream migrants from other subbasins and were subject to a density-dependent rearing stage (except Chinook salmon fry migrants, which only experienced density-independent productivity in the mainstem [11, 12]). Upon maturation, fish that moved out of their natal subbasin tributary and reared elsewhere were assumed to return as spawning adults to their natal subbasin tributary.

Based on the areas and qualities of each habitat type (Beechie et al. this volume), we developed individual habitat capacity and productivity parameters for Chinook and coho salmon, and steelhead populations in each subbasin. In some cases, a common set of parameters applied to all fish of a population in the basin (e.g., ocean maturation schedule, fecundity, estuary and ocean productivities). Freshwater life stage parameters were specifically determined for each of the 63 spatial units to capture the unique habitat characteristics of each subbasin for each population-specific life stage (e.g., spawning, incubation, and juvenile rearing capacities and productivities; current or natural potential; see S1 File).

The life stages were modeled as a sequence of either density-dependent or density-independent stages. Density-dependent stages used a Beverton-Holt function, applying the life stage capacities and productivities produced in the habitat analysis. The Beverton-Holt function used was:

$$N_{stage+1}=\frac{p\cdot N_{stage}}{1+\left(\frac{p}{c}\right)\cdot N_{stage}},$$ (1)

where *N*_*stage*_ is abundance of eggs or fish at the beginning of the stage, *p* is the productivity for the stage, *c* is the capacity for the stage, and *N*_*stage+*1_ is abundance of fish at the end of the stage (sensu [13]). Capacity is defined as the number of fish in a life stage that the habitat is able to support, which was calculated as the stage-specific density multiplied by habitat area which was scaled by the quality of the habitat (S2-S4 in S1 File). Productivity is life stage-specific and represents fecundity (reproductive stage) and survival, and is modified according to habitat conditions (S2-S4 in S1 File). The density-independent function was simply:

$$N_{stage+1}=p\cdot N_{stage},$$ (2)

with no capacity limit. The delta-bay and marine stages were all modeled as density-independent stages, with cumulative productivity rates through the bay and ocean adjusted such that modeled smolt-to-adult return (SAR) rates corresponded with the range of empirical observations and expert opinion (see Table 4 in S2 in S1 File). Annual marine productivity rates were taken from the literature, and we back-calculated the delta-bay productivity by dividing SAR by the annual ocean productivity rates weighted by the observed age structure of returning adults. The adult ocean maturity rate was fixed and did not vary.

LCM parameter values were determined through a combination of habitat quantity and quality estimates from the habitat analyses (Beechie et al. this volume), informed from literature values, and with input from local guidance about fish densities and productivities (S2 in S1 File). Habitat-derived capacities have been shown to be relatively close approximations to those estimated from empirical fish data [14]. Life cycle models often include some stochastic elements that introduce natural variability. However, we did not include stochasticity in the LCM parameters because we wanted to show potential effects on fish populations of particular habitat interventions.

## Salmon life histories

**Fig 2:**
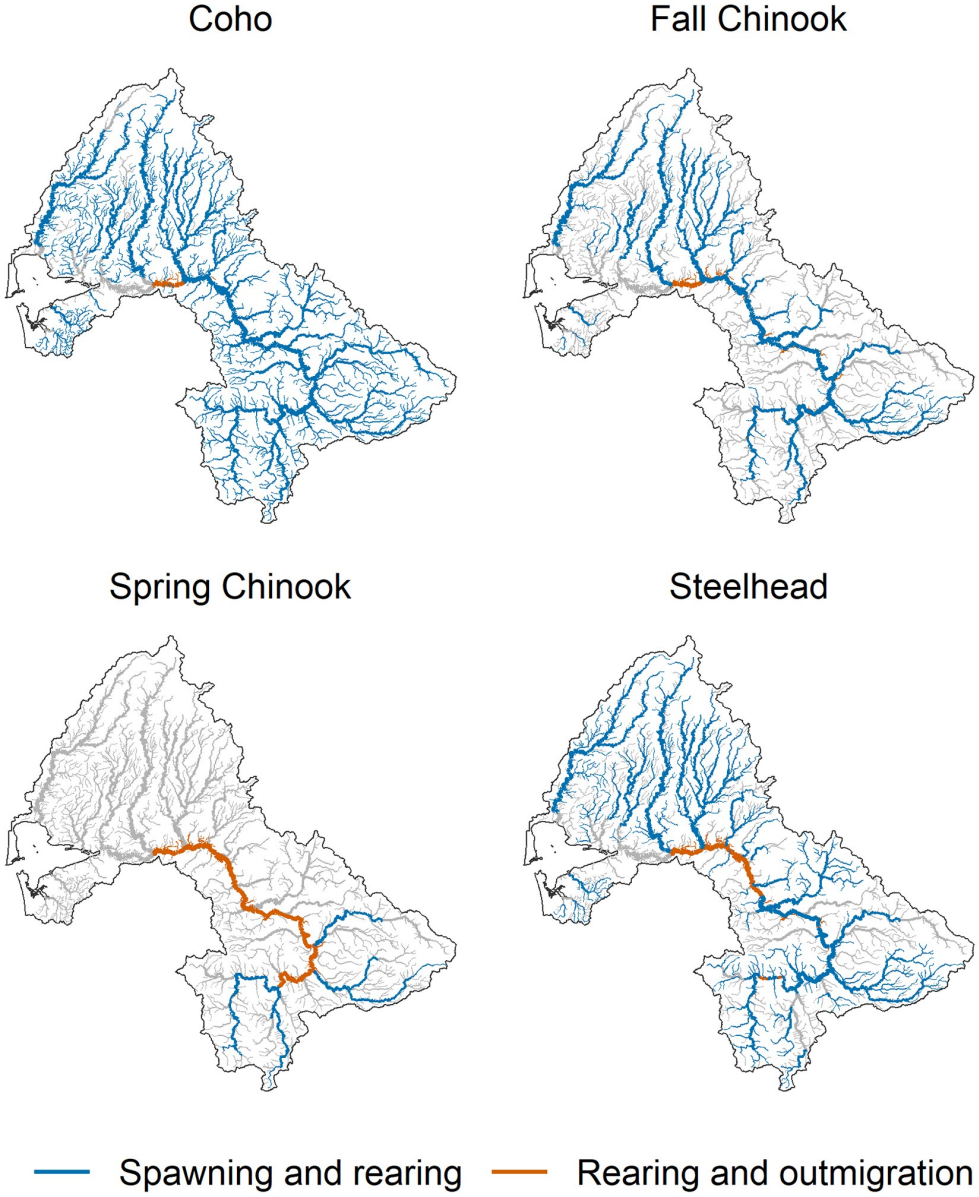
Spawning and freshwater rearing and outmigration reaches of four salmonid populations in the Chehalis River basin used in the life cycle models.

The following sections describe the structures of the LCMs we developed for each population. The number and structure of the life stages varied among populations (Table 1), as did the ranges of unique spawning and rearing habitats (Fig 2). More specific details of parameters can be found in S1 and S2 in S1 File.

**Table 1:** Summary of life stages and age classes included in the life cycle models for each population. X indicates that the life-stage and age class is included in the life cycle model for that population. Life stage parameters for each population and age class are described in S2 in S1 File, and habitat variables that modify those parameters are described in S3 in S1 File.

Parameter	Age	Coho	Spr. Chinook	Fall Chinook	Steelhead
Upstream migration	Varies	X	X	X	X
Spawning	Varies	X	X	X	X
Incubation	Age 0	X	X	X	X
Fry colonization	Age 0+	X	X	X	X
Fry outmigration	Age 0+	-	X	X	-
Subyearling rearing	Age 0+	-	X	X	-
1st year summer rearing	Age 0+	X	-	-	X
1st year winter rearing	Age 1	X	-	-	X
2nd year summer rearing	Age 1+	-	-	-	X
2nd year winter rearing	Age 2	-	-	-	X
3rd year summer rearing	Age 2+	-	-	-	X
3rd year winter rearing	Age 3	-	-	-	X
Delta-bay rearing	Fry migrant	-	X	X	-
	Subyearling	-	X	X	-
	Age 1	X	-	-	X
	Age 2	-	-	-	X
	Age 3	-	-	-	X
Ocean rearing	Age 1	-	X	X	-
	Age 2	X	X	X	X
	Age 3	X	X	X	X
	Age 4	-	X	X	X
	Age 5	-	X	X	X
	Age 6	-	-	-	X
Age at return	Age 2	X	X	X	-
	Age 3	X	X	X	X
	Age 4	-	X	X	X
	Age 5	-	X	X	X
	Age 6	-	X	X	X
	Age 7	-	-	-	X
Repeat spawning	Varies	-	-	-	X

### Coho salmon

Coho salmon enter the Chehalis River in October and November, and spawn from mid-October to December [15], and into January in the Satsop River (Curt Holt, WDFW, pers. comm.). Most juveniles rear in freshwater for about 18 months and leave as smolts in April and May. In small streams (bankfull width <20 m) in the summer, age 0 coho salmon generally rear in pool habitats [16] but in the winter they occupy stream pools and riffles at low densities and ponds or off-channel habitats at higher densities [17]. In larger rivers (bankfull width >20 m), age 0 juveniles occupy bank edge and backwater habitats in both the summer and winter [18]. Juvenile coho generally occupy velocities <45 cm/s with wood or plant cover in the summer, but in the winter they occupy velocities <15 cm/s and are most commonly associated with wood cover [18]. Coho spend approximately one year at sea and return to their natal river to spawn in their 3rd year [15].

The life cycle model for coho salmon has six freshwater life stages that were influenced by freshwater habitat conditions: adult upstream migration, spawning, egg incubation, fry colonization, summer rearing, and winter rearing. Upstream migration and spawning occur in the fall, eggs incubate during winter, and fry emerge in the spring. After fry emergence and colonization, fry experience a summer rearing period. A small percentage of fry move downstream from their natal subbasin in spring to rear in the mainstem Chehalis River. Another percentage of natal subbasin parr move downstream after the summer and rear in the mainstem over the winter period. Smolts then leave the basin, and experience emigration, delta-bay, and marine productivity. Data on the age structure of returning adults suggest that most adults return to spawn at age 3, with a small percentage of jacks returning at age 2.

### Spring and fall Chinook salmon

Chehalis River Chinook salmon are classified by the timing of adult river entry as either fall run (later fish arrival and spawn timing), or spring run (earlier fish arrival and spawn timing). Returning adults of the fall runs enter the Chehalis River from late August to mid-October and typically spawn in October and November [19]. For the spring run, adults return between late March and mid-July and spawning occurs between late August and early November [19]. Fry of both runs emerge from the gravel from February to June and migrate downstream either as fry migrants in a few weeks, or as sub-yearlings over a period of a few months. They primarily use edge and backwater habitats on their seaward migration [18]. Most subyearlings reach Grays Harbor between June and October [20]. Juvenile outmigrants rear in rivers in their first spring before migrating to salt water and adults rear at sea for two to five years before returning to spawn.

The spring and fall Chinook salmon LCMs have five freshwater life-stages that are influenced by freshwater habitat conditions: adult upstream migration, spawning, egg incubation, fry colonization, and subyearling rearing. Upstream migration productivity was a function of stream temperature for spring but not for fall run Chinook salmon. The remaining stages were modeled the same for both Chinook salmon populations (spring and fall). Fry exceeding the natal subbasin subyearling rearing capacity moved downstream through the mainstem to the delta-bay as fry migrants. Fry migrants were assumed to be in freshwater for two to four weeks as they moved to the delta-bay, and subyearling migrants were in freshwater for twelve weeks. Fry and subyearlings were assigned different productivity rates in the delta-bay and thereafter had similar ocean productivities. Most adults returning to spawn were ages 3 through 6 (S3 in S1 File).

### Steelhead

Adult spawning winter-run steelhead enter the Chehalis River Basin between December and May, and spawn from February through June [21]. Most juveniles rear in fresh water for 2 years and become outmigrating smolts ((3); G. Morishima, Technical Advisor to Quinault Indian Nation, personal communication). In small streams, juvenile steelhead do not exhibit strong habitat preferences, although there is a slight preference for low velocity backwater pools at age 0 [16]. In large rivers, age 0 juveniles occupy a wide range of edge habitat types and velocity classes in summer, but in winter they choose bank edge habitats with velocities <0.45 m/s [18]. Age 1 juveniles focus on bank edge habitats in both summer and winter, although velocity preferences are unclear [18]. Age at first spawning is typically 4 or 5 years for Chehalis River steelhead and repeat spawners are typically 5 to 7 years old ([21]; G. Morishima, personal communication; for more on repeat spawners, see below).

The life cycle model for steelhead had nine freshwater life stages that were influenced by freshwater habitat conditions: upstream migration, spawning, egg incubation, age 0+ summer rearing, age 0+ winter rearing, age 1+ summer rearing, age 1+ winter rearing, age 2+ summer rearing, age 2+ winter rearing. A percentage of age 1 parr moved downstream to the mainstem Chehalis River at the end of age 0+ summer rearing and again at the end of winter rearing. Some age 1 smolts left the basin to the estuary after the first winter. Age 2 smolts left the basin at the end of the second winter, and age 3 smolts left the basin at the end of the third winter rearing period. Smolts then experienced common delta-bay and marine productivities. Steelhead were the only species that had repeat spawners, with spawner ages ranging from 3 to 7 years. Repeat spawners represent adults that, after spawning, migrate back to the ocean and return the subsequent year to spawn again.

## Modeling life-stage capacities and productivities

Life stage capacities were estimated by multiplying end-of life stage fish densities for each habitat type by the total area and by multipliers based on the quality of a habitat type in tributaries in each subbasin (S2 in S1 File). Capacities of all habitat types for a given life stage were then summed at the subbasin spatial unit level (Fig 1), yielding a total life-stage capacity for each subbasin for each population and scenario. Changes in capacity could result from changes in habitat area or changes in quality (Beechie et al. this volume). Where there were empirical data to estimate changes in habitat areas from current to natural potential habitat conditions, the change in habitat area influenced the change in habitat capacity. In addition to changes in habitat area, a change in habitat quality can also influence capacity via a change in density. For example, an increase in stream temperature between the current and natural potential scenarios will reduce rearing capacity via a change in end-of-stage density (S4 in S1 File). The densities used for each population are in S2 in S1 File.

Productivity (or fecundity in the case of the spawning life stage) was a function of either habitat type or habitat quality (or population in the case of fecundity; S2 in S1 File). Empirical data from the literature often indicated that different habitat types have different productivity values. For example, over-winter productivities for coho salmon are relatively low in tributary channels (mean survival = 0.35, [22]), but much higher in beaver ponds (mean survival = 0.78, [22]). In these cases, the estimated life stage productivity for a subbasin tributary was calculated as the average of the two productivities, weighted by the capacity of each habitat type within a tributary. To evaluate changes in habitat quality across the scenarios (see scenarios section below), we calculated a difference in productivity or created a productivity multiplier as a function of the change in the habitat quality attribute (S3 in S1 File). For example, incubation productivity was calculated as a function of percent fine sediment, whereas summer rearing productivity was modified as a function of stream temperature. There were no local data and few literature sources to inform delta-bay productivities. However, we had local guidance for SAR and literature values for annual ocean productivities. Because SAR is the product of the delta-bay and annual ocean productivities, we back-calculated delta-bay productivity for each population by dividing SAR by the annual ocean productivities.

During life cycle model development, we frequently compared model outputs to limited information related to observed population spawner abundances and estimated age structure of juveniles and adults where available (basin-wide and for specific subbasins) to assess model performance. When model outputs were substantially different from observations, we checked parameters and code for errors and made adjustments based on re-examination of data or literature. This process included examination of outputs of capacity, productivity, and abundance at the end of each life stage or by subbasin, so that we could focus error-checking efforts on the most likely parameters and functions driving model outputs.

The LCMs were initialized for a period of 10 years to seed the models with abundances for all life stages, and after a model burn-in of 100 years results are reported for the subsequent 100-year model run period during which an equilibrium abundance was reached.

## Development of diagnostic scenarios

The purpose of the diagnostic scenarios was to help understand the relative restoration potential in each type of degraded habitat for each population. Using the natural potential and estimated current life-stage capacities and productivities, we developed a current condition scenario (used for building the models) and several comparative diagnostic habitat scenarios. The current conditions scenario set all habitats to estimated current conditions, where habitat parameters were estimated under the commonly occurring seasonal flow conditions (S2 in S1 File). The diagnostic scenarios began with all current conditions and then set one habitat component at a time to natural potential conditions to help determine which types of habitat losses most constrain recovery of salmon and steelhead populations in each subbasin. The diagnostic scenarios evaluated the separate influences of:

1. Migration barriers
2. Fine sediment in spawning gravels
3. Wood abundance change in small streams and large rivers
4. Shade (temperature) changes in small streams and large rivers
5. Large river channel length and bank condition
6. Beaver pond changes in small streams
7. Floodplain habitat change (including side channels, ponds, marshes, and lakes)

Each habitat change affected one or more life stage parameter for one or more population (summarized in Tables 2 and 3). Details of the functional relationships used in the LCMs to estimate changes in life stage capacity or productivity for each habitat attribute are in S3 in S1 File.

**Table 2:** Life stage capacities (*c*) and productivities (*p*) affected by each habitat factor in the habitat model (Beechie et al. this volume) and life-cycle models for coho salmon and steelhead. The value *c*_*egg*_ is egg capacity, *p*_*incub*_ is incubation productivity, *c*_*sr*_ is summer rearing capacity, *p*_*sr*_ is summer rearing productivity, *c*_*wr*_ is winter rearing capacity, and *p*_*wr*_ is winter rearing productivity.

	*c*_*egg*_	*p*_*incub*_	*c*_*sr*_	*p*_*sr*_	*c*_*wr*_	*p*_*wr*_
Barriers	X		X^1^	X	X^1^	X
Fine sediment		X				
Wood loading	X		X	X	X	X
Shade			X	X		
Large river	X		X	X	X	X
Beaver pond area	X^2^		X	X	X	X
Floodplain			X	X	X	X

^1^Effect expressed only when barrier is 100% blocking.

^2^Negative effect.

**Table 3:** Life stage capacities (*c*) and productivities (*p*) affected by each habitat factor in the habitat model (Beechie et al. this volume) and life-cycle models for spring and fall Chinook. The value *p*_*prespawn*_ is prespawn productivity, *c*_*egg*_ is egg capacity, *p*_*incub*_ is incubation productivity, *c*_*sub*_ is subyearling rearing capacity, and *p*_*sub*_ is subyearling rearing productivity.

	*p*_*prespawn*_	*c*_*egg*_	*p*_*incub*_	*c*_*sub*_	*p*_*sub*_
Barriers		X		X^1^	X
Fine sediment			X		
Wood loading		X		X	X
Shade	X^2^			X	X
Large river		X		X	X
Beaver pond area		X^3^		X	X
Floodplain				X	X

^1^Effect expressed only when barrier is 100% blocking.

^2^Spring Chinook salmon only.

^3^Negative effect.

## Results

**Fig 3:**
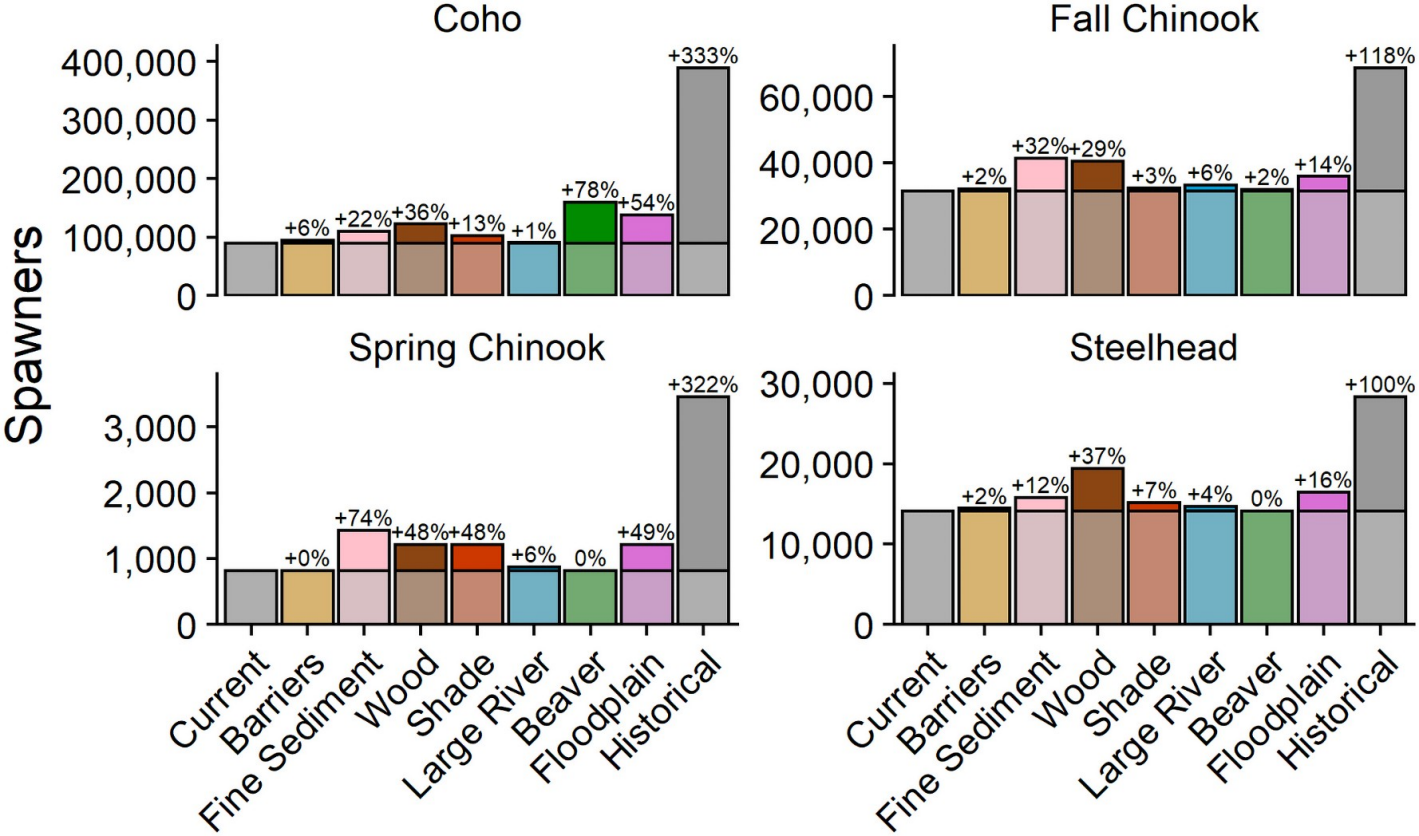
Spawner abundances in response to diagnostic scenarios that estimated freshwater habitat changes relative to the estimated current abundance. The brighter intensity colors above the line, which correspond to estimated current conditions, are shown along with percent change from current conditions.

**Fig 4:**
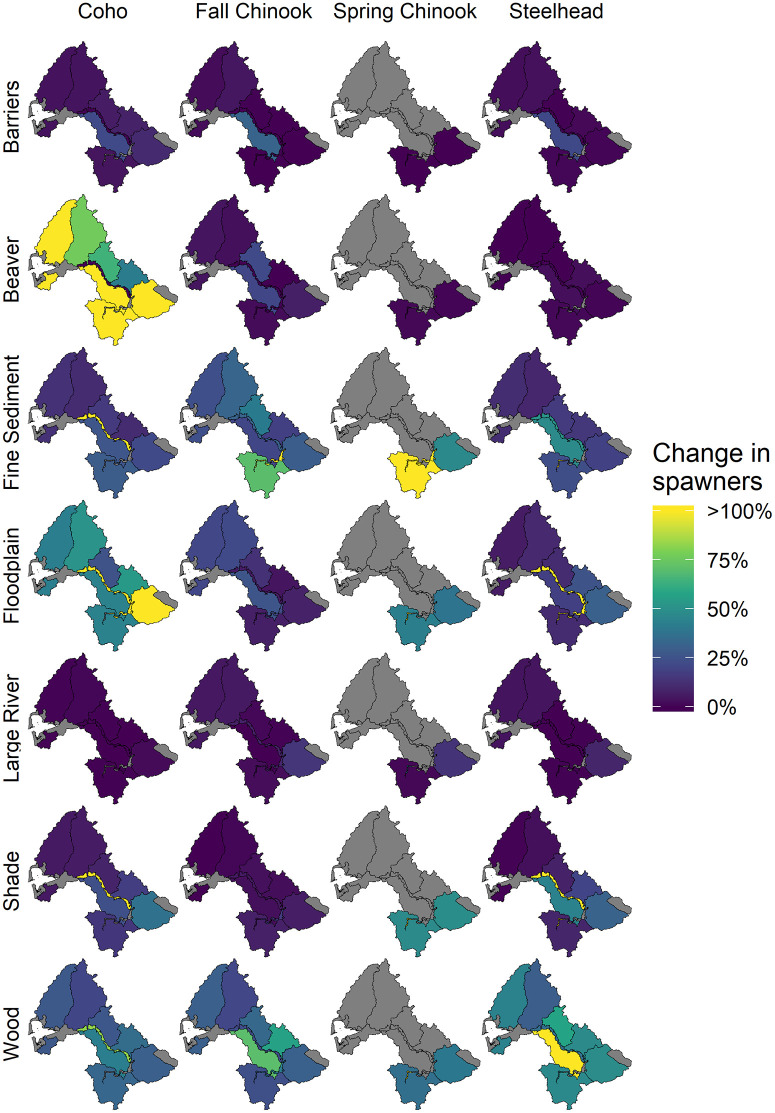
Percent change in spawner abundance resulting from each of the diagnostic scenarios of habitat change. Colors represent the percent change in spawner abundance in each Ecological Region and scenario relative to the abundance of the Ecological Region in the current condition. Ecological Regions in gray have no spawners.

The basin-level results compared effects of different types of habitat restoration, as shown by the potential to increase salmon populations at the scale of Chehalis basin, but the magnitude of restoration potential varied spatially, as did the distributions of populations within the basin. Hence, we present results for both scales of analysis: basin-wide (Fig 3) and Ecological Region-level (Fig 4).

## Basin-wide habitat effects

Our results indicated that increases in salmon abundance under each diagnostic scenario varied among populations at the scale of the Chehalis River basin (Fig 3). Scenarios produced widely varying increases in spawner abundance, reflecting differences in habitat preferences and sensitivities among populations. In the beaver pond scenario, coho salmon spawner abundance increased by 76% but other populations showed little increase in abundance. The floodplain scenario increased coho salmon abundance by 54% and spring Chinook salmon by 40%. Fall Chinook salmon and steelhead spawner abundances increased 14–15% in the floodplain scenario.

The wood abundance scenario produced moderate increases in spawner abundance for all four populations (29–45% increase). In the shade scenario, only spring Chinook salmon abundance increased substantially with a projected 37% increase. Coho abundance increased by only 10% and both fall Chinook salmon and steelhead were relatively insensitive to change in shade. Finally, spring and fall Chinook salmon were sensitive to changes in fine sediment in spawning gravels (63% and 32% increase, respectively), whereas coho and steelhead were less responsive (21% and 11% increase, respectively).

Removal of migration barriers produced a 6% or less increase in spawner abundance for all populations.

## Spatial distribution (Ecological Region-level) of habitat effects

For coho and fall Chinook salmon, percentage increases in spawner abundance within the same Ecological Regions relative to current conditions were similar (generally 20–40%) (Fig 4). For spring Chinook salmon, the percent increase in spawner abundance was highest in the Mainstem Upper Chehalis Ecological Region (>100%), but the absolute abundance increase was low because of the relatively low abundance in this region. Most of the modeled increase in spring Chinook salmon spawner abundance in the natural potential wood scenario was in the Cascade Mountains Region (in primarily two subbasin tributaries, Skookumchuck River and Newaukum River). For steelhead in the wood scenario, spawner abundance increased ~25% up to ~120% in all Ecological Regions except the mainstem regions.

Percent change in coho salmon spawner abundance under the floodplain habitat scenario was high across all Ecological Regions except for the mainstem regions (Fig 4). In the mainstem Chehalis River there were no estimated spawner abundance changes because there was no spawning occuring in those reaches. Increased survival of juveniles from improved mainstem habitat was included in the respective natal Ecological Regions from which juveniles migrated. For coho, floodplain habitat is important for the overwinter life stage, whereas for spring Chinook salmon, floodplain connectivity is most important for temperature reductions during the prespawn life stage and for increasing spawning capacity through addition of side channel length. Because fall Chinook salmon are less dependent on floodplain habitats, percent increases in spawner abundance were generally low. For steelhead, the floodplain scenario produced increases of <30% in spawner abundance, except in the lower mainstem Chehalis where increased side channel length increased spawner capacity significantly (>100%), but because there were a small number of spawners in the mainstem the total increase was small.

The beaver pond scenario produced very large spawner abundance increases for coho salmon for all Ecological Regions except the mainstem areas (Fig 4). Beaver ponds are a preferred winter rearing habitat for coho salmon, and estimated juvenile survival through the winter is considerably higher in beaver ponds than in stream channels. There were very small increases in spawner abundance for fall Chinook salmon in the Olympic Mountains and Grays Harbor Ecological Regions. Spring Chinook salmon and steelhead showed very little potential response to increased beaver pond habitat area.

The shade scenario produced a relatively small basin-wide change in coho salmon spawner abundance (10%) despite high summer stream temperatures in the Chehalis basin. This is because the stream temperature change from current to natural potential shade was near 0°C in most Ecological Regions, and less than 2°C in much of the remaining area. However, a few tributary Ecological Regions had relatively large percentage changes in coho spawner abundance because current shade conditions are locally very poor, notably the Cascade Mountains Ecological Region (with an increase of ~40%; Fig 4). Spring Chinook salmon showed large percent increases in spawner abundance in the shade scenario in the Cascade Mountains, Willapa Hills, and Upper Mainstem Chehalis Ecological Regions. In these three regions, current stream temperatures are substantially higher compared to estimated natural potential conditions within holding and spawning reaches for spring Chinook salmon. Therefore, the shade scenario produced at least an approximate 40% increase in each location. However, the Upper Mainstem Chehalis has very few spawners. Fall Chinook salmon are less sensitive to temperature changes because they enter the river after high summer temperatures subside and, consequently, the shade scenario produced increases in spawner abundance of less than ~10% in all Ecological Regions. Juvenile steelhead have a higher thermal tolerance than coho salmon, and the shade scenario showed only small increases in steelhead spawner abundance.

The overall response of coho salmon was small for the diagnostic scenario with barriers removed (6% increase; Fig 3), indicating that the removal of barriers had a relatively small impact on coho salmon at the scale of the entire Chehalis River basin. However, the Central Lowlands Ecological Region had a 20–30% increase in spawner abundance for coho and both Chinook salmon populations. Individual barrier removals had locally larger impacts when viewed at the subbasin and tributary scale. This indicates that barrier removal is an important restoration opportunity for coho salmon in some locations but that, overall, a small proportion of coho habitat is blocked to adult migration. There are no identified migration barriers in the range of spring Chinook salmon, so there was no response of spring Chinook salmon in the barrier removal scenario. Fall Chinook salmon and steelhead are exposed to a few barriers, but there were no significant localized or basin-wide impacts.

There was at least a ~10% or greater increase in abundance at the Ecological Region-level in response to the changes in fine sediment for all populations across the Chehalis basin (Fig 4). Increases in productivity were moderate to high across all populations and Ecological Regions. However, capacity increases were generally low because fine sediment affects a density-independent life stage. Outside of the mainstem reaches where spawner abundance was low, the largest increase was in the Willapa Hills (25–100% increase). Modeled changes in fine sediment were based on forest road density (Beechie et al. this volume) and resulted in relatively large potential increases in incubation productivity parameters for each population. Percent change in spawner abundance under the fine sediment scenario was most pronounced for spring and fall Chinook salmon and steelhead, and was somewhat lower for coho salmon. There was high uncertainty in both the predicted fine sediment levels in the sediment model as well as in identification of sediment sources.

## Discussion

In our process-based HARP model, we first quantified changes in key habitat-forming processes and habitat attributes from their natural potential (Beechie et al. this volume), and in these next steps of the HARP model, we used life-cycle models to evaluate which degraded conditions (e.g., riparian vegetation, floodplain availability, etc.) represent the largest restoration opportunities for each population and spatial unit [1]. Each diagnostic scenario evaluated one causal mechanism of habitat change, which equates to one potential restoration action type. The diagnostic scenarios suggested that loss of floodplain habitat, wood from streams and rivers, riparian shade, and beaver ponds most constrain population abundances in the Chehalis basin (Table 4). While the modeling also indicates that all populations are sensitive to fine sediment effects on incubation survival, more work is needed to identify specific locations and sources of high fine sediment to reduce the uncertainty of its effect. Migration barrier removals are locally important potential restoration actions, but removal of barriers will likely have small effects on basin-wide spawner abundance of all populations. Restoring main channel length and removing bank armor are unlikely to measurably improve spawner abundance of any population.

**Table 4:** Summary of potential restoration actions based on the diagnostic scenario results.

Restoration action	Summary of restoration potential
Floodplain reconnection	Reconnection of floodplain habitats provides overwintering habitat for coho salmon, as well as decreasing stream temperature and increasing side channel spawning and rearing areas for Chinook salmon and steelhead. Among subbasins, the Skookumchuck, Newaukum, Black, Humptulips, Wynoochee, and Satsop rivers have large floodplain restoration potential. The lower mainstem Chehalis River also has significant floodplain habitat restoration potential.
Wood placement	Wood restoration is likely to modestly benefit all populations. Larger habitat changes are likely in small, moderate-slope reaches where wood substantially increases pool and spawning gravel area. The potential benefits of wood restoration are relatively evenly distributed across the subbasins, and the analysis does not indicate strong spatial priorities for wood restoration.
Riparian shade restoration	Riparian restoration includes both riparian planting and protection. It is likely to significantly increase shade and reduce stream temperature in a few areas, some of which are very important to spring Chinook salmon. The largest restoration potentials are in the Skookumchuck, Newaukum, and Black rivers, and other small tributaries to the lower mainstem Chehalis River.
Beaver pond restoration	Restoring beaver ponds to small streams is likely to significantly benefit coho salmon (more than doubling the population in the historical beaver pond scenario), with relatively small effects on the other three populations. The potential for recovery of beaver ponds and beaver populations is highest in subbasins with a high proportion of low-gradient small streams.
Barrier removal	Whereas the potential for barrier removals to benefit populations is small overall, local benefits can be larger (e.g., in subbasins such as the Skookumchuck, Cloquallum, Newaukum, and South Fork Chehalis).
Fine sediment reduction	The diagnostic scenario for historical fine sediment indicates that spring and fall Chinook salmon subpopulations are very sensitive to fine sediment levels, however, we are unsure of where and what types of restoration actions are needed. This suggests that field assessments of fine sediment levels and sources of fine sediment should be conducted to identify the most important sources of sediment to address through restoration actions.
Large river	The diagnostic scenario in the large rivers included restoring length and removing bank armoring. This action is unlikely to measurably improve spawner abundance of any population. Chinook salmon use the large rivers for both spawning and rearing and had modest potential in the Skookumchuck and Newaukum.

Importantly, the HARP model approach does not focus on construction of lost habitats, but on restoring the key mechanisms that create those habitats [23], such as restoring floodplain connectivity, riparian functions for wood recruitment and shade, or beaver populations to create beaver pond habitat. However, in the near term some habitat creation actions such as wood placement or constructing beaver dam analogs [24, 25] are important strategies for recovery of salmon because of the long lag time between riparian tree growth and recovery of wood abundance or beaver populations.

## Summary of restoration options

### Floodplain restoration and wood abundance

**Fig 5:**
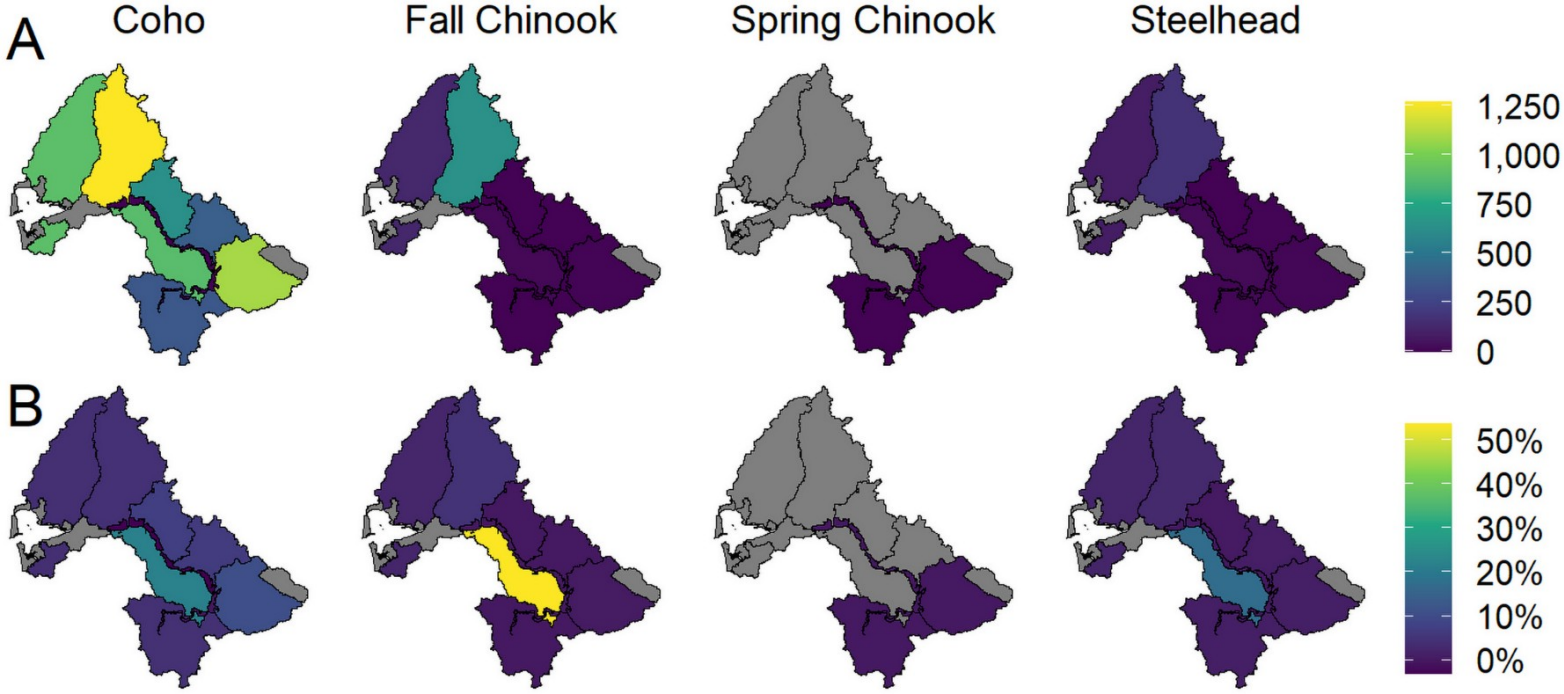
Spatially-explicit potential for spawner abundance to increase as a result of floodplain habitat restoration, presented as absolute increases from current conditions (top row) and as percent change (bottom row) relative to current Ecological Region-specific abundances. Ecological Regions in gray have no spawners.

The diagnostic scenarios indicated that restoring floodplain habitat and wood abundance is likely to significantly benefit all four populations, with floodplain restoration most benefiting coho salmon and spring Chinook salmon, and wood restoration most benefitting spring and fall Chinook salmon and steelhead. Importantly, results for specific tributary subbasins and for each population indicated that floodplain habitat restoration in the lower mainstem (from Skookumchuck River downstream to Wynoochee River) will increase multiple subpopulations of coho upstream of the Wynoochee River, and also improve spring and fall Chinook salmon populations to a lesser degree. Among Ecological Regions, the Cascade Mountains, Olympic Mountains and Grays Harbor Tributaries have large floodplain restoration potential, both when ranked by absolute abundance and by percent increase (Fig 5). Each of those areas had significant historical marsh habitat that has been lost or degraded (Beechie et al., this volume). Only the Black River Ecological Region has an appreciable portion of its historical marsh remaining today. By contrast, the potential benefits of wood restoration were more evenly distributed across the subbasins, and the analysis did not indicate strong spatial priorities for wood restoration. However, the scientific literature generally indicates that wood restoration in small, moderate-slope reaches has the greatest potential to increase pool area, which benefits multiple populations that occupy those reach types (primarily coho salmon and steelhead; [26, 27]).

### Beaver pond restoration

**Fig 6:**
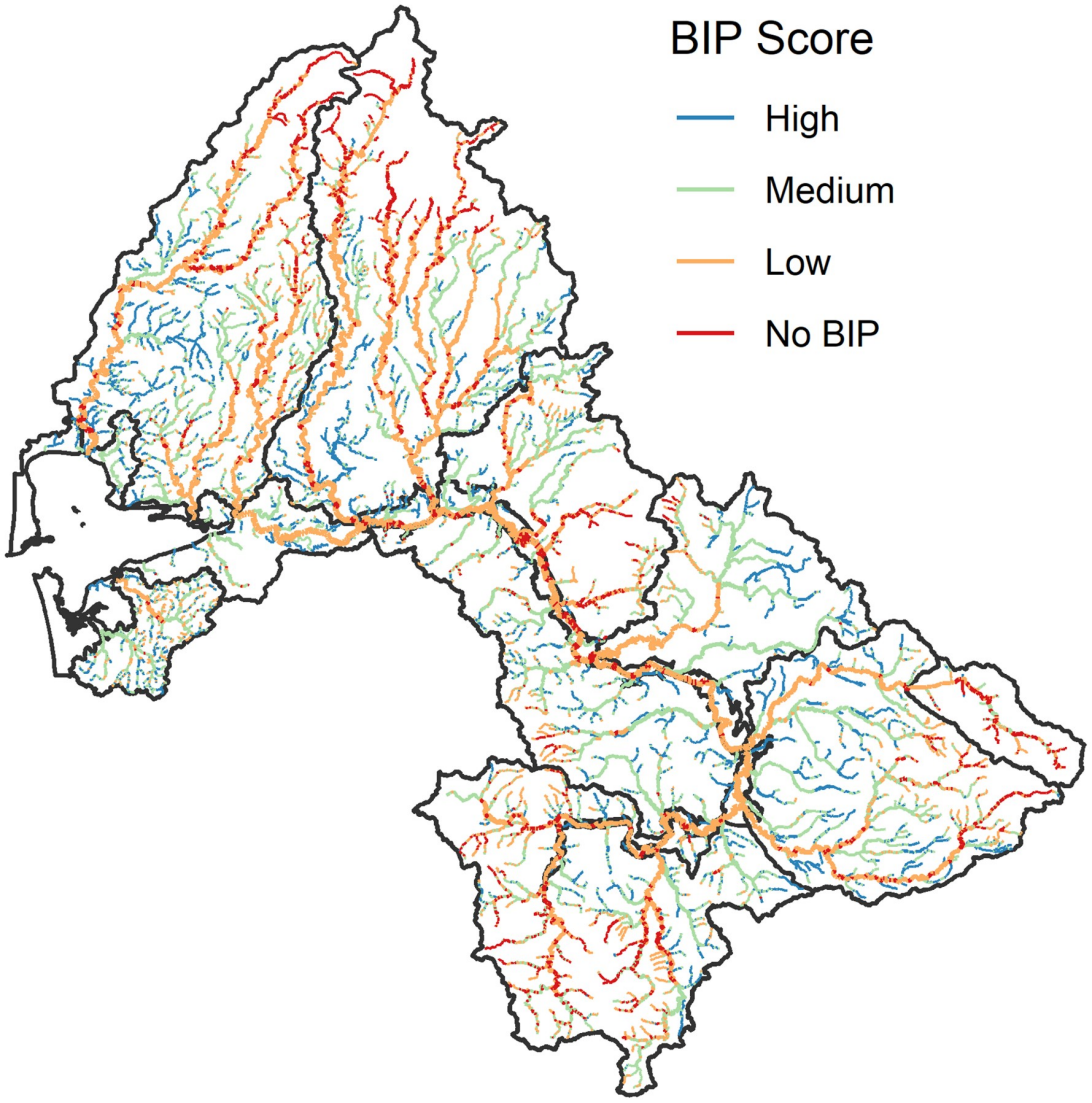
Map of beaver intrinsic potential in the Chehalis River basin, based on a modified version of the beaver intrinsic potential model of Dittbrenner et al. (2018).

Restoring beaver ponds to small streams is likely to significantly benefit coho salmon (more than doubling the population in the natural potential beaver pond scenario), with relatively small effects on the other three populations. The potential for recovery of beaver ponds and beaver populations is greatest in small, low-slope channels with wide valleys [28]. We modified an existing method for estimating the intrinsic potential of habitats to support beavers [28] (Table 5) and used this to estimate beaver restoration potential for the most suitable locations within the range of coho salmon in the Chehalis basin (Fig 6). In general, areas with lower potential are in the upper Olympic Mountains, Black Hills, Cascade Mountains, and Willapa Hills Ecological Regions, which are the four areas with predominantly volcanic lithology and steeper streams. Areas of alluvium, glacial deposits, and marine sedimentary rocks all contain significant low-slope stream length with high or medium beaver intrinsic potential, and are therefore good candidates for beaver reintroductions [28].

**Table 5:** Scoring system for beaver intrinsic potential, modified from Dittbrenner (2018).

Stream slope and score		Stream width and score		Cumulative Score	BIP category
<1%	4	<7 m	4	7–8	High
1–2%	3	7–10 m	3	6	Medium
2–4%	2	10–18 m	2	4–5	Low
4–6%	1	18–24 m	1	<4	No BIP
6–10%	0.5				
>10%	0	>24 m	0		

### Riparian restoration

Riparian restoration is likely to significantly increase shade and reduce stream temperature in a few areas, some of which are very important to spring Chinook salmon. The natural potential shade scenario indicated that reduction of stream temperature in spring Chinook salmon holding and rearing areas can potentially double the spring Chinook population under the current climate, and increase coho abundance by 13%. The comparison of current to natural potential shade levels in the Chehalis basin showed that more than 60% of the basin has riparian shade conditions that are currently near their natural potential, mostly occurring inside the Olympic National Forest or state and private managed forests (Beechie et al. this volume). Much of that stream length has a modeled temperature difference of <0.5°C, indicating very little potential for continued tree growth to improve temperature conditions in the future (Beechie et al. this volume). Areas with temperature change >2°C are mostly concentrated in the Cascade Mountains Ecological Region, and to a lesser extent in the Black River, Willapa Hills, and Lower and Middle Mainstem Chehalis Ecological Regions.

### Barrier removal

**Fig 7:**
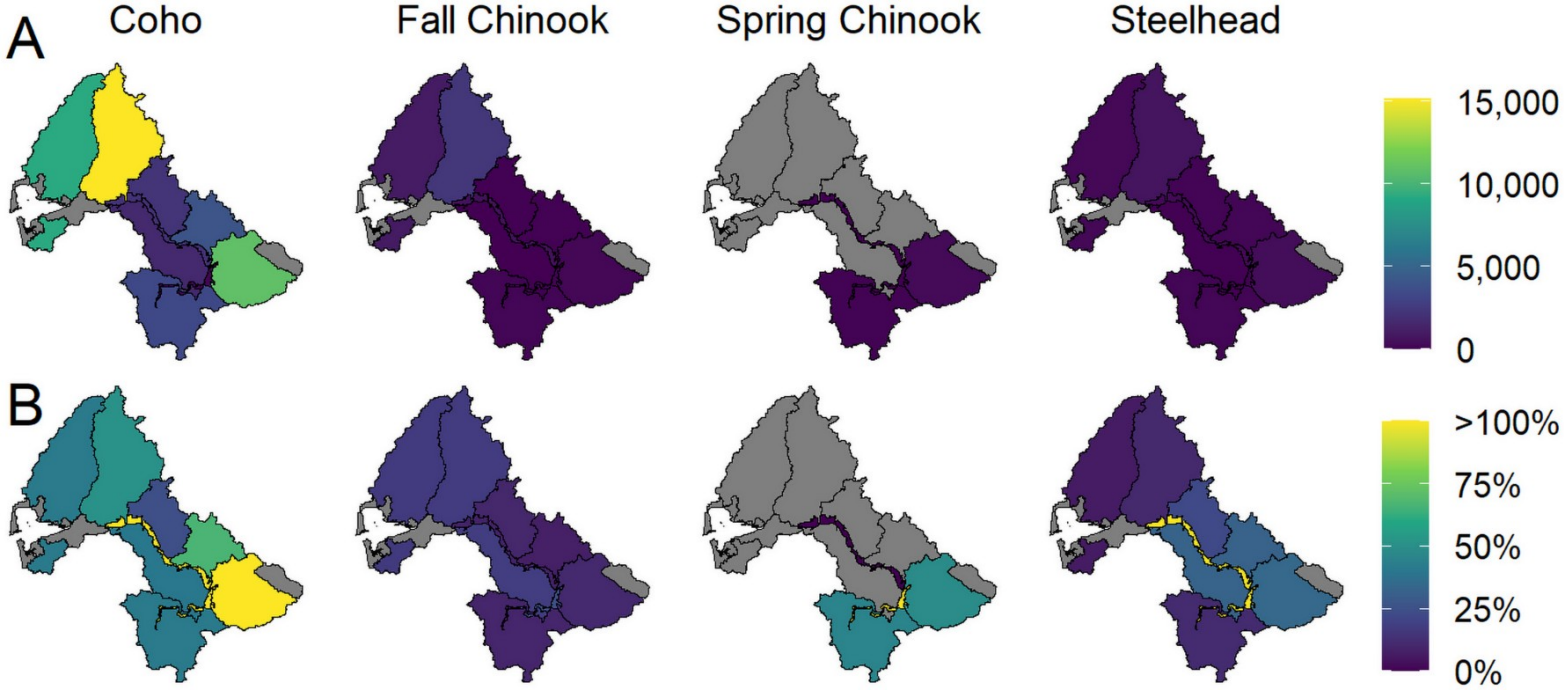
Spatially-explicit potential for spawner abundance to increase as a consequence of barrier removal, represented by absolute increases from current conditions (top row) and as percent change (bottom row) relative to current Ecological Region-specific abundances. Ecological Regions in gray have no spawners.

Although the potential for barrier removals to benefit populations is small overall (especially for spring Chinook salmon, which have no migration barriers within their range), there are specific subbasin tributaries in which barrier removals can significantly improve local subpopulations of coho salmon (Fig 7), and modestly improve subpopulations of fall Chinook and steelhead. The no-barrier diagnostic scenario indicated that barrier removals or passage improvements should provide the largest percentage increases in coho salmon abundance in the small tributaries to the mainstem from the middle to upper Chehalis River, but the largest potential absolute abundance increases were in the Olympic and Cascade Mountains Ecological Regions. While barrier removals were not likely to provide the largest abundance increases among scenarios for any population, local benefits can be large and potentially cost-effective to achieve. The primary uncertainty in the barriers scenario was in the reported barrier passability ratings as percent reductions in capacity and productivity. A study directed at verifying the passability ratings system with observed estimates of the ability of fish to pass the barriers would reduce uncertainty in barrier effects.

### Fine sediment reduction

The diagnostic scenario for fine sediment indicated that there is considerable potential to improve Chinook salmon subpopulations (and to a lesser extent, steelhead) by reducing fine sediment levels in spawning gravels. However, the model of fine sediment is based on data relating forest roads to fine sediment levels, with no other land uses considered (Beechie et al. this volume). Moreover, there are very few empirical observations of fine sediment in the Chehalis basin to confirm that fine sediment levels are high relative to natural conditions.

## Variability, stochasticity, and uncertainty

Natural variability and parameter uncertainty are often included in life cycle models as stochastic components, but in this study we chose not to employ stochastic elements in the results for ease of comparison across diagnostic scenarios. However, we acknowledge uncertainties in the models, including: model uncertainty (accuracy of model form), parameter uncertainty (accuracy of parameter estimates, including measurement error and extrapolation error), scenario uncertainty (uncertainty in modeling future development or climate change effects), data uncertainty that was used to fit models, and natural variability (natural annual variation) [29–31]. We intend to address some of these uncertainties in future work.

Substantial local knowledge gaps contribute to model form uncertainty. For example, we do not know the extent to which model results are affected by inter- and intra-population interactions in the subbasins, delta, and bay, nor do we understand the exchange of individuals between reaches within or across tributaries [32]. These interactions, as well as predation by native and non-native species, may have influenced life cycle modeling outcomes had we included them, and may have also influenced the potential success of restoration actions. For example, reconnection of a floodplain habitat that has abundant predators may not increase salmon populations as expected because the benefit of increased capacity is negated by decreased productivity. Also, we do not know how natural fish production is affected by hatchery supplementation (e.g., [33]). While such interactions may be important, we do not have local data to incorporate such effects, and they were beyond the scope of this study.

The reach-level habitat values used in the model contain varying types and levels of parameter uncertainty (see Beechie et al. this volume for more details). For measured parameters such as large river bank habitat or riparian canopy opening angle, the main source of uncertainty is measurement error because these parameters are not extrapolated or modeled. In contrast, reach-level habitat values such as percent pool area in small streams were extrapolated from a sample of field surveys. We attempted to limit extrapolation error by stratifying the data by channel slope and adjacent land cover, and then extrapolating data to reaches in the same slope and land cover class. Finally, we may have introduced prediction error by borrowing certain habitat parameters from other models (e.g., the fine sediment values discussed earlier).

Another source of parameter uncertainty is that we used various literature sources to estimate spawning and rearing densities or productivities to parameterize current conditions in the LCMs. We chose productivity values from the higher end of the observed ranges to avoid producing abundances that were biased low and ensured that the LCMs generated fish production closer to the theoretical capacity as estimated from the habitat analyses Beechie et al. (this volume). In contrast, using the mean of published productivity estimates could have led to underestimation of potential fish production in the basin. For estimating capacities, we used published densities by habitat type (e.g., [34]), assuming that those densities represented a high estimate of current observed densities. In other cases, such as with the large river densities, we chose the 95th percentile of empirical densities from published studies to calculate capacity, also attempting to assure that capacity estimates—and therefore fish abundances—were not biased low and lead to underestimates of population sizes from the LCMs.

## Conclusions

This study highlights the usefulness of a coupled habitat-LCM approach like HARP to inform and prioritize process-based restoration activities. In the first step (Beechie et al. this volume), determining the causes of habitat degradation and establishing the benchmark of natural conditions illustrated the potential for restoration. Using that information in a life cycle modeling framework identified not only action types but also the locations for targeting population-specific efforts that would potentially benefit fish the most, and it also suggested areas that require further investigation. The model also elucidated important differences in habitat restoration needs among species, showing that restoration actions must address sensitive life-stages for each species to substantially increase their abundance. Basins with substantial amounts of local fish data may gain the most benefit from coupled habitat-LCM applications like this because model outputs can be checked for consistency with biological data.

The HARP model is a flexible modeling approach that can address a variety of habitat changes for which current conditions can be compared to some estimate of the natural potential condition, and the number and types of restoration actions or processes addressed using this model can be tailored to local needs and data availability. Examples of habitat factors that could be added include the roles of food resources and estuary habitat changes, each of which may elucidate other population constraints or habitat restoration options. Ultimately, whether few or many habitat-forming process and conditions are addressed, this modeling approach quantifies how process relationships control the ways in which habitat changes drive salmon abundance, and provides a means of valuing restoration potential in the context of species of interest. In this way, the HARP modeling approach provides an understanding of how each type of restoration opportunity is likely to affect abundance of key species, and restoration planners and policy makers can set priorities for those restoration actions that most benefit targeted species or are most cost effective. When combined with clear understanding of the causes of habitat change, restoration plans can focus on root causes of degradation and identify key habitat-forming processes to restore.

## Supporting information

S1 File (DOCX)

## References

[1] Beechie T, Pess G, Morley S, Butler L, Downs P, Maltby A, et al. Watershed Assessments and Identification of Restoration Needs. In: Stream and Watershed Restoration. John Wiley & Sons, Ltd; 2013. p. 50–113.

[2] Beechie T, Pess G, Roni P, Giannico G. Setting River Restoration Priorities: A Review of Approaches and a General Protocol for Identifying and Prioritizing Actions. North Am J Fish Manag. 2008 Jun;28(3):891–905.

[3] Beechie T, Bolton S. An Approach to Restoring Salmonid Habitat-forming Processes in Pacific Northwest Watersheds. Fisheries. 1999;24(4):6–15.

[4] Bartz K, Lagueux K, Scheuerell M, Beechie T, Haas A, Ruckelshaus M. Translating restoration scenarios into habitat conditions: An initial step in evaluating recovery strategies for Chinook salmon (Oncorhynchus tshawytscha). Can J Fish Aquat Sci—CAN J Fish AQUAT SCI. 2006 Jul 1;63:1578–95.

[5] Scheuerell MD, Hilborn R, Ruckelshaus MH, Bartz KK, Lagueux KM, Haas AD, et al. The Shiraz model: a tool for incorporating anthropogenic effects and fish–habitat relationships in conservation planning. Can J Fish Aquat Sci. 2006 Jul 1;63(7):1596–607.

[6] Steel E, Fullerton A, Caras Y, Sheer M, Olson P, Jensen D, et al. A Spatially Explicit Decision Support System for Watershed-Scale Management of Salmon. Ecol Soc [Internet]. 2008 Dec 11 [cited 2020 Jul 23];13(2). Available from: https://www.ecologyandsociety.org/vol13/iss2/art50/main.html

[7] Fullerton AH, Steel EA, Lange I, Caras Y. Effects of Spatial Pattern and Economic Uncertainties on Freshwater Habitat Restoration Planning: A Simulation Exercise. Restor Ecol. 2010 Nov;18:354–69.

[8] Zabel RW, Scheuerell MD, McClure MM, Williams JG. The Interplay between Climate Variability and Density Dependence in the Population Viability of Chinook Salmon. Conserv Biol. 2006;20(1):190–200. doi: 10.1111/j.1523-1739.2005.00300.x 16909672

[9] Pess G, Jordan C, Armour M, Beechie T, Bond M, Cooney T, et al. Characterizing Watershed-Scale Effects of Habitat Restoration Actions to Inform Life Cycle Models: Case Studies Using Data-Rich vs. Data-Poor Approaches. NMFS-NWFSC; 2019 Dec. Report No.: 151.

[10] Greene, Correigh M., Beechie TJ. Habitat-specific population dynamics of ocean-type chinook salmon (Oncorhynchus tshawytscha) in Puget Sound. Can J Fish Aquat Sci. 2004;61:590–602.

[11] Anderson JH, Topping PC. Juvenile Life History Diversity and Freshwater Productivity of Chinook Salmon in the Green River, Washington. North Am J Fish Manag. 2018;38(1):180–93.

[12] Zimmerman MS, Kinsel C, Beamer E, Connor EJ, Pflug DE. Abundance, Survival, and Life History Strategies of Juvenile Chinook Salmon in the Skagit River, Washington. Trans Am Fish Soc. 2015 May 4;144(3):627–41.

[13] Moussalli E, Hilborn R. Optimal Stock Size and Harvest Rate in Multistage Life History Models. Can J Fish Aquat Sci. 1986;43:135–41.

[14] Bond MH, Nodine TG, Beechie TJ, Zabel RW. Estimating the benefits of widespread floodplain reconnection for Columbia River Chinook salmon. Can J Fish Aquat Sci. 2019;76(7):1212–26.

[15] Weitkamp, Laurie A., Wainwright TC, Bryant GJ, Milner GB, Teel DJ, Kope RG, et al. Status Review of Coho Salmon from Washington, Oregon, and California. National Marine Fisheries Service; 1995. Report No.: 24.

[16] Bisson PA, Sullivan K, Nielsen JL. Channel Hydraulics, Habitat Use, and Body Form of Juvenile Coho Salmon, Steelhead, and Cutthroat Trout in Streams. Trans Am Fish Soc. 1988;117(3):262–73.

[17] Brown TG, Hartman GF. Contribution of Seasonally Flooded Lands and Minor Tributaries to the Production of Coho Salmon in Carnation Creek, British Columbia. Trans Am Fish Soc. 1988;117(6):546–51.

[18] Beechie TJ, Liermann M, Beamer EM, Henderson R. A Classification of Habitat Types in a Large River and Their Use by Juvenile Salmonids. Trans Am Fish Soc. 2005 May;134(3):717–29.

[19] Myers J, Kope R, Bryant G, DJ T, Lierheimer L, Wainwright T, et al. Status Review of Chinook Salmon From Washington, Idaho, Oregon, and California. NMFS-NWFSC; 1998 Jan p. 443. Report No.: 35.

[20] Sandell T, Fletcher J, McAninch A, Wait M. Grays Harbor Estuary Salmonid Conservation and Restoration Plan [Internet]. Wild Fish Conservancy; http://wildfishconservancy.org/projects/grays-harbor-juvenile-salmon-fish-community-study/WFC2015GraysHarborEstuaryconservationplan.final.pdf

[21] Busby PJ, Wainwright TC, Bryant, Gregory J., Lierheimer LJ, Waples, Robin S., Waknitz FW, et al. Status Review of West Coast Steelhead from Washington, Idaho, Oregon, and California. NMFS-NWFSC; 1996 p. 16. Report No.: 27.

[22] Ogston L, Gidora S, Foy M, Rosenfeld J. Watershed-scale effectiveness of floodplain habitat restoration for juvenile coho salmon in the Chilliwack River, British Columbia. Can J Fish Aquat Sci. 2014 Nov 13;72(4):479–90.

[23] Beechie TJ, Sear DA, Olden JD, Pess GR, Buffington JM, Moir H, et al. Process-based Principles for Restoring River Ecosystems. BioScience. 2010 Mar;60(3):209–22.

[24] Pollock MM, Beechie TJ, Wheaton JM, Jordan CE, Bouwes N, Weber N, et al. Using Beaver Dams to Restore Incised Stream Ecosystems. BioScience. 2014 Apr 1;64(4):279–90.

[25] Roni P, Beechie T, Pess G, Hanson K. Wood placement in river restoration: fact, fiction, and future direction. Jonsson B, editor. Can J Fish Aquat Sci. 2015 Mar;72(3):466–78.

[26] Montgomery DR, Buffington JM, Smith RD, Schmidt KM, Pess G. Pool Spacing in Forest Channels. Water Resour Res. 1995 Apr;31(4):1097–105.

[27] Beechie TJ, Sibley TH. Relationships between channel characteristics, woody debris, and fish habitat in northwestern Washington streams. Trans Am Fish Soc. 1997;126(2):217–29.

[28] Dittbrenner BJ, Pollock MM, Schilling JW, Olden JD, Lawler JJ, Torgersen CE. Modeling intrinsic potential for beaver (Castor canadensis) habitat to inform restoration and climate change adaptation. Munderloh UG, editor. PLOS ONE. 2018 Feb 28;13(2):e0192538. doi: 10.1371/journal.pone.0192538 29489853 PMC5831098

[29] Francis R, Shotton R. “Risk” in fisheries management: a review. Can J Fish Aquat Sci. 1997 Aug 1;54(8):1699–715.

[30] Rosenberg AA, Restrepo VR. Uncertainty and Risk Evaluation in Stock Assessment Advice for U.S. Marine Fisheries. Can J Fish Aquat Sci. 1994 Dec 1;51(12):2715–20.

[31] Steel E, Liermann M, McElhany P, Scholz N, Cullen A. Managing uncertainty in habitat recovery planning. In: Ecosystem recovery planning for listed salmon: assessment approaches for salmon habitat. NMFS-NWFSC; 2003. p. 74–89. (NOAA Technical Memorandum; vol. 58).

[32] Rieman BE, Dunham JB. Metapopulations and salmonids: a synthesis of life history patterns and empirical observations. Ecol Freshw Fish. 2000;9(1–2):51–64.

[33] Christie MR, Ford MJ, Blouin MS. On the reproductive success of early-generation hatchery fish in the wild. Evol Appl. 2014 Sep;7(8):883–96. doi: 10.1111/eva.12183 25469167 PMC4211718

[34] Nickelson TE. A Habitat-Based Assessment of Coho Salmon Production Potential and Spawner Escapement Needs for Oregon Coastal Streams. In 1998.

