# Supplementary material for: Identifying the potential of anadromous salmonid habitat restoration with life cycle models
Source: PLoS One. 2021 Sep 9;16(9):e0256792. doi: 10.1371/journal.pone.0256792 (PMC8428657; doi:10.1371/journal.pone.0256792)
Supplement: S1 File — (DOCX) [file pone.0256792.s001.docx]

# Supplemental Information for “Identifying the potential of salmon habitat restoration with life cycle models”

Jeffrey C Jorgensen^1^, Colin Nicol^2^, Caleb Fogel^2^, Timothy J. Beechie^1^

^1^ Fish Ecology Division, Northwest Fisheries Science Center, National Marine Fisheries Service, National Oceanic and Atmospheric Administration, Seattle, Washington, United States of America

^2^ Ocean Associates, Inc., Under contract to Northwest Fisheries Science Center, National Marine Fisheries Service, National Oceanic and Atmospheric Administration, Seattle, Washington, United States of America

# S1. Life Cycle Model Descriptions

For each population modeled (coho, spring and fall Chinook salmon, steelhead), we describe the structure of the life-cycle model, and briefly describe the calculation of capacity and productivity parameters for each life stage.

## Coho salmon

The life cycle model for coho salmon has six freshwater life stages (upstream migration, spawning, egg incubation, fry colonization, summer rearing, and winter rearing) that are influenced by freshwater habitat conditions (Figure S1-1). After both fry emergence and summer rearing a portion of the juveniles redistribute to the mainstem. Smolts leave the basin after winter rearing and experience emigration, delta-bay, and marine productivity. Calculations of capacities and productivities are described in Table S1-1.


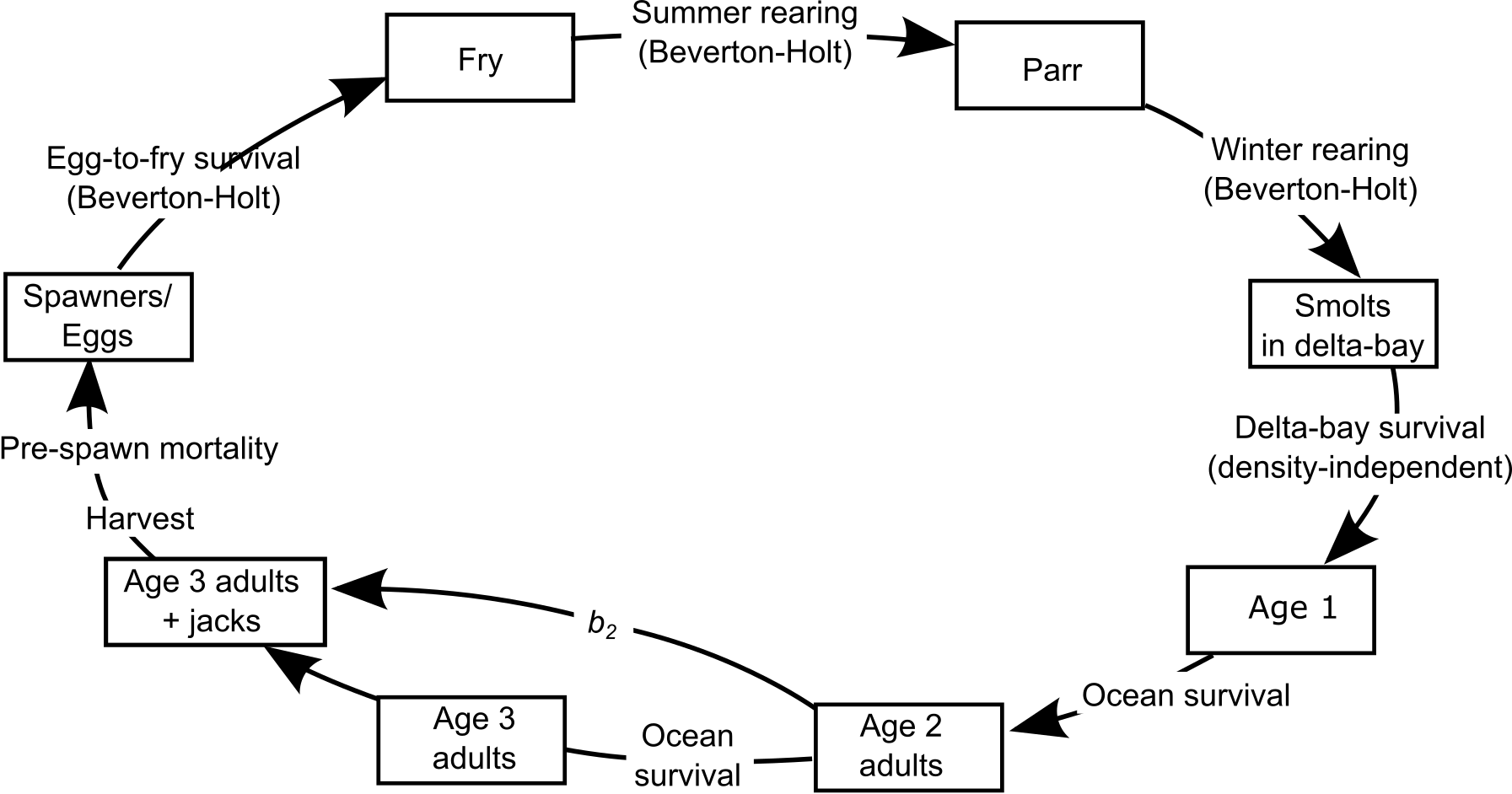


Figure S1-1. Schematic diagram of the life-cycle model for coho salmon in the Chehalis River basin.

| Table S1-1. Overview of coho salmon life stages, and factors affecting productivity, capacity, and redistribution. Productivity, *p*, and area, *A*, can be either fixed or a function of habitat type, *h*. Habitat quality multipliers (*β*) impact productivity and density. Calculation of *A* is described in Beechie et al. (this volume) and *β* is described in S4. The reach-level weights (w) for productivities are based on spawning or rearing capacities for a habitat type (*w_spawn_* = spawning capacity weight, *w_summer_* = summer rearing capacity weight, *w_winter_* = winter rearing capacity weight).   \| **Life Stage** \| **Productivity** \| **Capacity** \| **Redistribution** \| \| --- \| --- \| --- \| --- \| \| Prespawn \| $0.95 \beta_{imperv} \beta_{passage} w_{spawn}$ \| ∞ \| --- \| \| Spawning \| 2500 \| $A_{h}d_{h}\beta_{passage}$ \| --- \| \| Incubation \| ${\beta_{sed} \beta}_{passage} w_{spawn}$ \| ∞ \| --- \| \| Fry colonization \| 0.68 \| ∞ \| 0.05 \| \| Summer rearing \| ${p_{h} \beta}_{t,coho}\beta_{wood} w_{summer}$ \| ${A_{h}d_{h} \beta}_{t,coho}\beta_{wood}$ \| $f(c_{winter})$ \| \| Winter rearing \| $p_{h}\beta_{wood} w_{winter}$ \| $A_{h}d_{h}\beta_{wood}$ \| --- \| \| SAR \| 0.04 \| --- \| --- \| |  |  |  |
| --- | --- | --- | --- | --- | --- | --- | --- | --- | --- | --- | --- | --- | --- | --- | --- | --- | --- | --- | --- | --- | --- | --- | --- | --- | --- | --- | --- | --- | --- | --- | --- | --- | --- | --- | --- |

## Spring and fall Chinook salmon

The spring and fall Chinook models have five freshwater life stages that are influenced by freshwater habitat conditions (upstream migration, spawning, egg incubation, fry colonization, and subyearling rearing) (Figure S1-2). In the models, fry colonize natal subbasin rearing habitats first and fry exceeding the natal subbasin rearing capacity move downstream through the mainstem to the bay as fry migrants. Fry migrants are assumed to be in freshwater for one week in their natal basin, and subyearling migrants are in freshwater for twelve weeks (Mara Zimmerman, WDFW, personal communication). Fry migrants have additional migration mortality, but that mortality is absorbed in the delta-bay productivity value in the model. Fry and subyearlings are assigned different productivity rates in the delta-bay and, thereafter, have similar ocean productivities. Because of the different productivities in the delta-bay, fry and subyearling migrants have different smolt-to-adult return rates. A description of the calculations and parameters for the life stages is shown in Table S1-2.


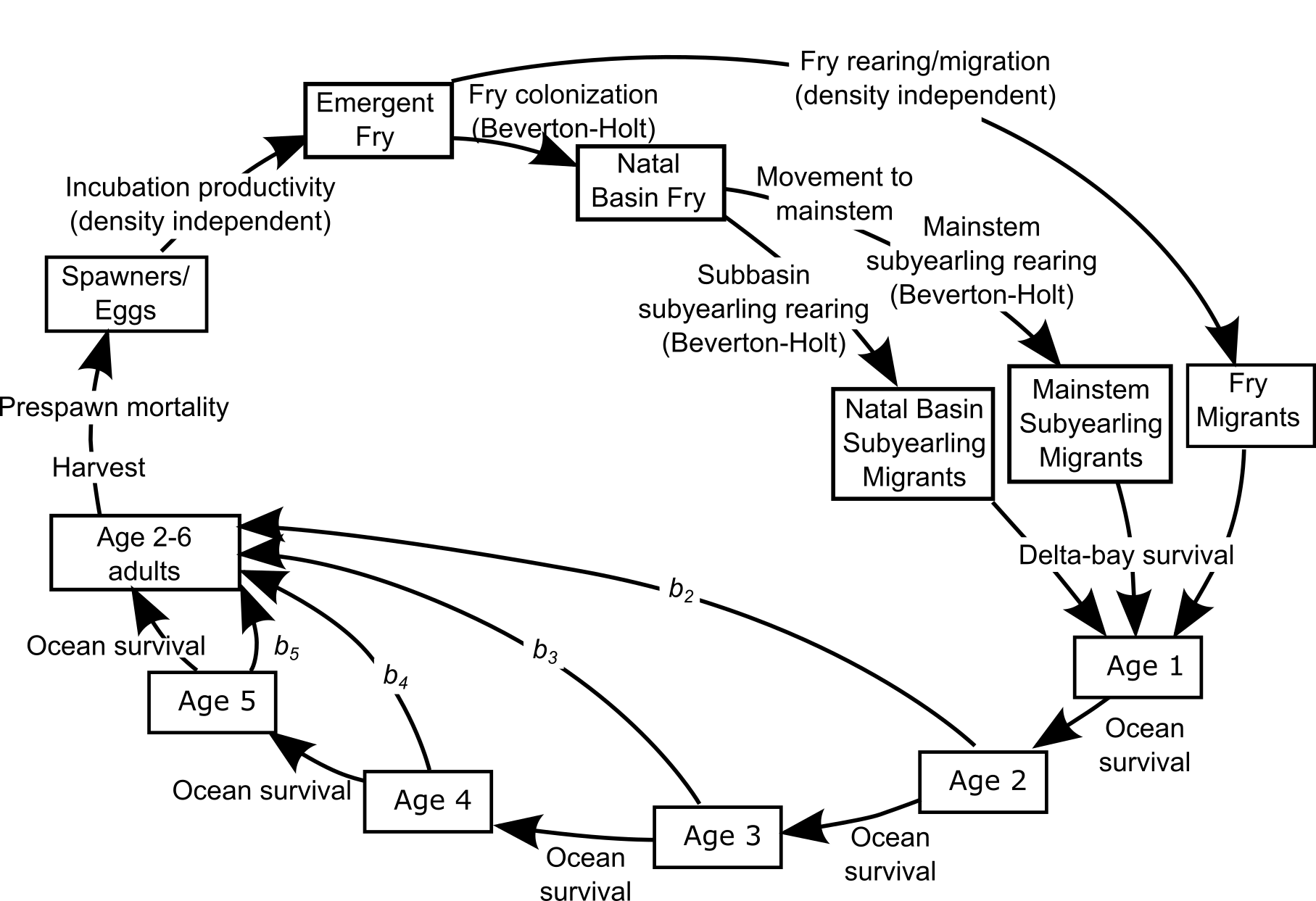


Figure S1-2. Schematic diagram of the life-cycle model for spring and fall Chinook salmon in the Chehalis River basin.

Table S1-2. Overview of Chinook salmon life stages. Productivity, *p*, and density, *d*, can be either fixed or a function of habitat type *h*. Habitat quality multipliers (*β*) impact productivity and density. Spawning capacity for Chinook salmon is based on spawning area. Calculation of *A* is described in Beechie et al. (this volume) and *β* is described in S4 ^*^ $\beta_{t,ps}$ is used for spring Chinook only and omitted for fall Chinook. The reach-level weights (w) for productivities are based on spawning or rearing capacities for a habitat type (*w_spawn_* = spawning capacity weight, *w_fry_* = fry colonization capacity weight, *w_subyr_* = subyearling rearing capacity weight).

| **Life Stage** | **Productivity** | **Capacity** | **Redistribution** |
| --- | --- | --- | --- |
| Prespawn | \| $\beta_{passage} \beta_{t,ps}*w_{spawn}$ \| \| --- \| | ∞ | --- |
| Spawning | 5400 | \| $A_{h}d_{h}\beta_{passage}$ \| \| --- \| | --- |
| Incubation | \| ${\beta_{sed} \beta}_{passage} w_{spawn}$ \| \| --- \| | ∞ | --- |
| Fry colonization | \| $p_{h}\beta_{wood} w_{fry}$ \| \| --- \| | \| $A_{h}d_{h}\beta_{wood}$ \| \| --- \| | $f$(density dependence) |
| Subyearling rearing | \| ${p_{h} \beta}_{t,chino}\beta_{wood} w_{subyr}$ \| \| --- \| | \| ${A_{h}d_{h} \beta}_{t,chino}\sigma_{wood}$ \| \| --- \| | --- |
| SAR | Dependent on proportions of fry and subyearling migrants to the estuary (see Fig S1-3) | | --- |


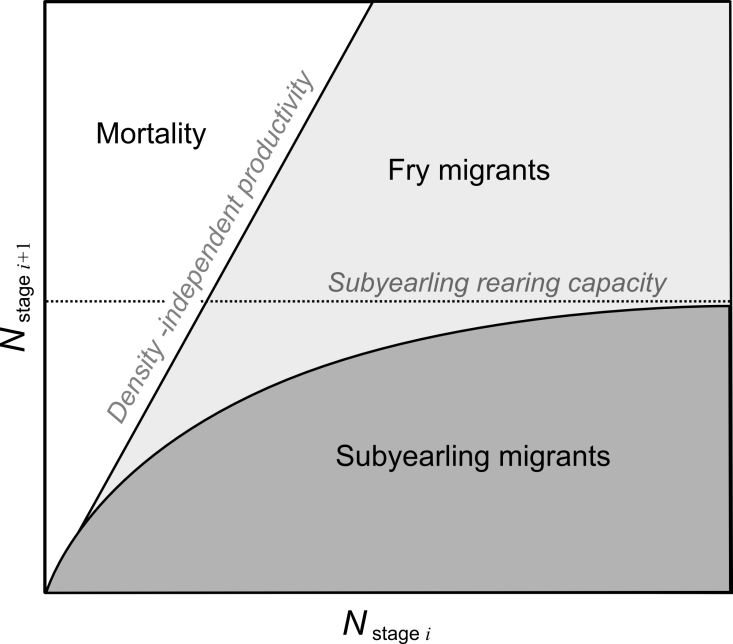


Figure S1-3. Illustration of the Beverton-Holt density-dependent movement calculations in the spring and fall Chinook models. Fry between the Beverton-Holt curve and the density independent productivity line (light gray) leave the river in the first week as fry migrants. Fry below the Beverton-Holt curve (dark gray) stay in the natal subbasin for one week and then redistribute throughout the natal basin and downstream mainstem reaches to become subyearling migrants. Adapted from Greene and Beechie (2004).

## Steelhead

The life cycle model for steelhead has nine freshwater life stages that are influenced by freshwater habitat conditions: upstream migration, spawning, egg incubation, age-0+ summer rearing, age-0+ winter rearing, age-1+ summer rearing, age-1+ winter rearing, age-2+ summer rearing, and age-2+ winter rearing (Figure S1-4). Because some age-1 parr move down to the mainstem at the end of the first summer and first winter (not shown in diagram), age-1+ and-2+ parr are split into natal and mainstem groups. Smolts then leave the basin and experience delta-bay and annual marine productivity. An overview of the life stages and how capacities and productivities are calculated is shown in Table S1-3. A complete list of parameter values is in S2.


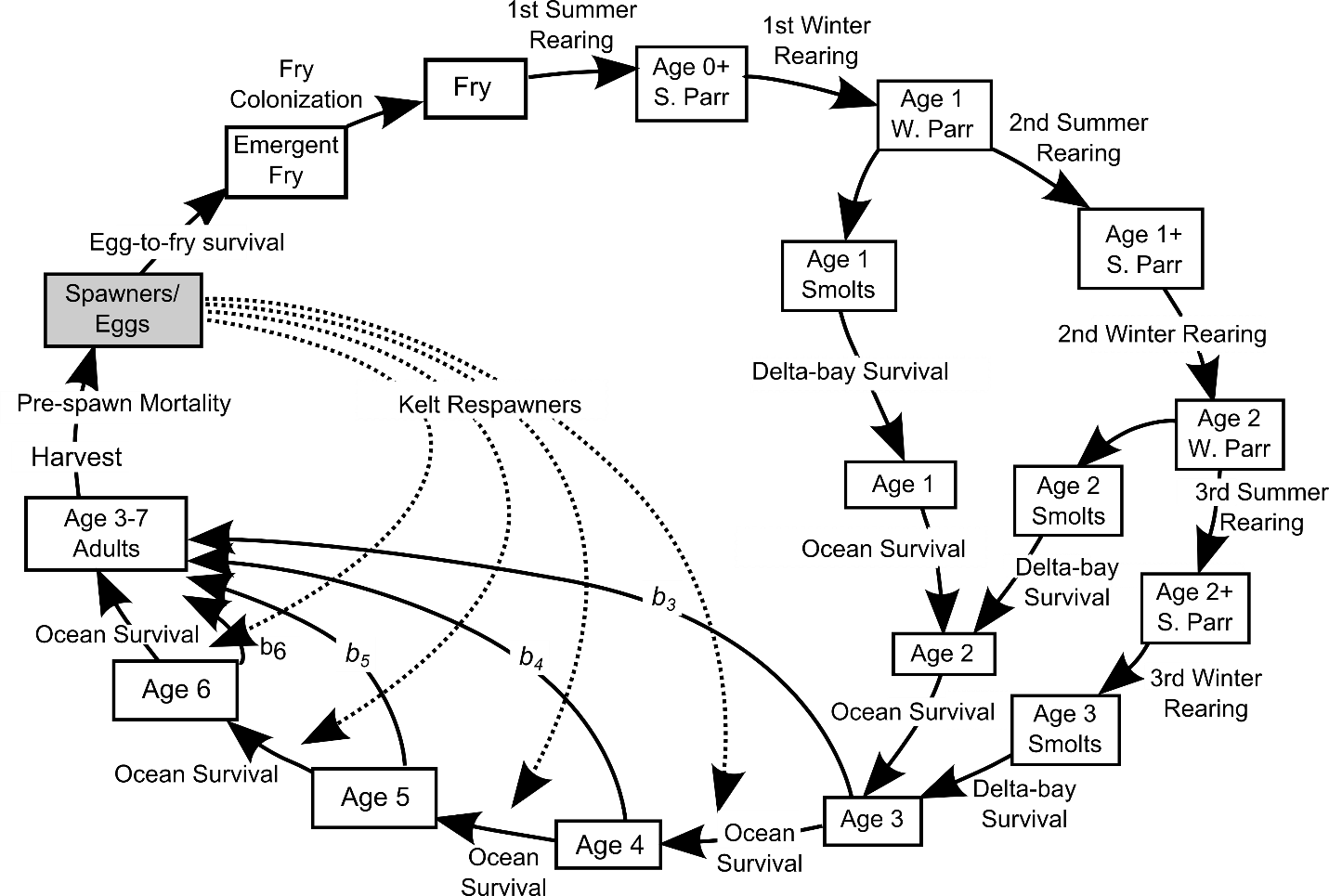


Figure S1-4. Schematic diagram of the life-cycle model for steelhead in the Chehalis River basin.

Table S1-3. Overview of steelhead life stages. Productivity, *p* and area, *A*, can be either fixed or a function of habitat type, *h*. Habitat quality multipliers (*β*) impact productivity and density. Calculation of *A* is described in Beechie et al. (this volume) and *β* is described in S4. The reach-level weights (w) for productivities are based on spawning or rearing capacities for a habitat type (*w_spawn_* = spawning capacity weight, *w_summer_* = summer rearing capacity weight, *w_winter_* = winter rearing capacity weight).

| **Life Stage** | **Productivity** | **Capacity** | **Redistribution** |
| --- | --- | --- | --- |
| Prespawn | $0.95 \beta_{imperv} \beta_{passage} w_{spawn}$ | ∞ | --- |
| Spawning | 5400, 8000 | $A_{h}d_{h}\beta_{passage}$ | --- |
| Incubation | ${\beta_{sed} \beta}_{passage} w_{spawn}$ | ∞ | --- |
| Summer rearing - age 0+ | ${p_{h} \beta}_{t,sthd}\beta_{wood} w_{summer0+}$ | ${A_{h}d_{h} \beta}_{t,sthd}\beta_{wood}$ | $f$(subbasin area) |
| Winter rearing - age 1 | $p_{h}\beta_{wood} w_{winter1}$ | $A_{h}d_{h}\beta_{wood}$ | $f$(subbasin area) |
| Summer rearing - age 1+ | ${p_{h} \beta}_{t,sthd}\beta_{wood} w_{summer1+}$ | ${A_{h}d_{h} \beta}_{t,sthd}\beta_{wood}$ | --- |
| Winter rearing - age 2 | $p_{h}\beta_{wood} w_{winter2}$ | $A_{h}d_{h}\beta_{wood}$ | --- |
| Summer rearing - age 2+ | ${p_{h} \beta}_{t,sthd}\beta_{wood} w_{summer2+}$ | ${A_{h}d_{h} \beta}_{t,sthd}\beta_{wood}$ | --- |
| Winter rearing - age 3 | $p_{h}\beta_{wood} w_{winter3}$ | $A_{h}d_{h}\beta_{wood}$ | --- |
| SAR | 0.08 | ∞ | --- |

## Redistribution

Redistribution in the model is defined as fish movement from a natal tributary spatial unit downstream to a mainstem unit to rear (Figure S1-5). After redistribution, fish from several natal basins can comingle and increase each other’s density dependent mortality. There are two methods for redistribution. The first method is short redistribution, where juveniles move down stream to the first mainstem reach they encounter and rear in that reach. The second method is long redistribution, where juveniles move to the mainstem and spread evenly between all reaches from the first reach they encounter down to the delta.


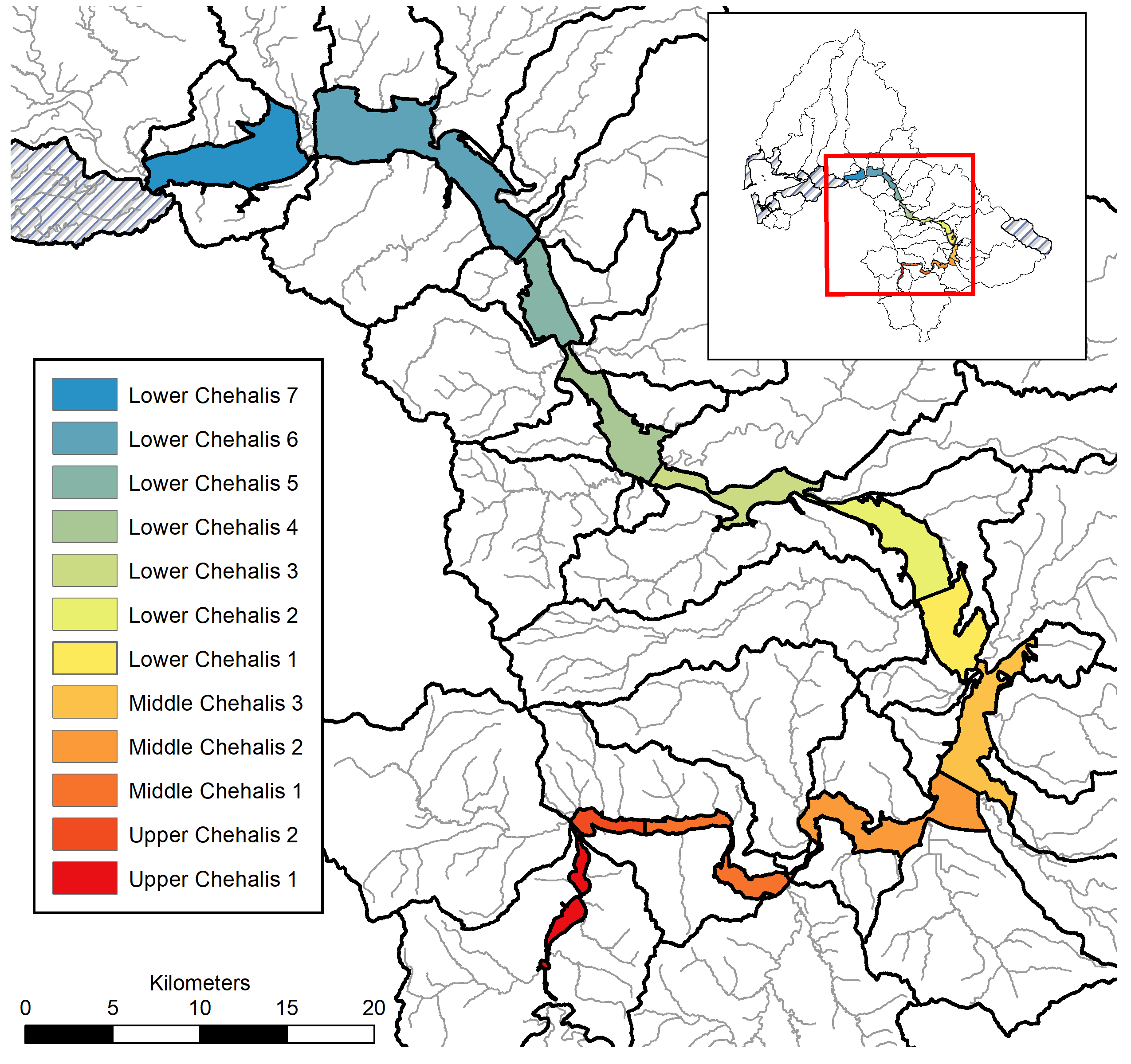


Figure S1-5: Mainstem Chehalis River sections to which some fish redistribute during the freshwater rearing stages.

### Coho redistribution in the spring

After spring fry emergence, juvenile coho redistribute using the short redistribution method. A fixed proportion of 5% of juveniles from every natal tributary unit move down to the first mainstem unit they encounter.

### Coho redistribution in the fall

After summer rearing, a proportion of coho juveniles redistribute by moving downstream to the first mainstem unit they encounter. They then spread evenly downstream in the mainstem down to the section just before where the delta/bay begins. The proportion of fish in each natal basin that redistribute to the mainstem is a function of the quality of winter habitat through the habitat assessments (Beechie et al. this volume). The proportions of fish moving downstream was informed from input from the Chehalis Basin Scientific Review Team. In subbasins with poor winter habitat conditions more fish move downstream and in subbasins with good winter habitat conditions fewer fish move downstream to mainstem units. Under current conditions, in subbasins with the poorest winter habitat, 11% of juveniles move down to the mainstem. Under historical wood conditions, 7% of juveniles move downstream. With historical floodplain conditions, 3% of fish redistribute to the mainstem.

### Chinook subyearling redistribution

Subyearling Chinook salmon use the mainstem to rear as they are outmigrating. After the fry colonization stage, subyearlings use the long redistribution method to move to the first mainstem unit they encounter and spread evenly throughout the mainstem to the delta.

### Steelhead Age-0+ and Age-1 redistribution

After rearing for their first summer, age-0+ steelhead redistribute downstream but do not move into the nearest mainstem unit; instead, they remain in their natal subbasin. Therefore, their movements were not explicitly modeled and thus the habitat parameters for their natal subbasin apply.

After rearing over their first winter, a fraction of age-1 steelhead redistribute downstream to the mainstem. Redistribution percentage is inversely related to natal basin area such that 0% of juveniles move from basins larger than 450 km^2^, 10% of juveniles move from basins between 150-450 km^2^ and 50% of juveniles move in natal basins smaller than 250 km^2^.

# S2. Calculation of Life Stage Capacity and Productivity

For each life stage listed in S1, a capacity and productivity value is calculated for each spatial unit. The process to calculate capacity is habitat area multiplied by fish density and scaled by habitat quality. Productivity is simply the baseline productivity (described below) for a given life stage scaled by habitat quality. In cases where density and productivity vary by habitat type, subbasin capacity is the sum of all habitat-specific capacities and productivity is the simple average productivity weighted by the habitat-specific capacity. Density independent stages are the average productivity of all habitat units within a spatial unit.

The following equations show the calculation of the habitat quality multiplier, capacity, and productivity for one spatial unit. Because both capacities and productivities are modified by the habitat quality multipliers, we describe the calculation of the multipliers first, followed by capacity and productivity.

## Habitat Quality Multipliers

Habitat quality scales both life stage productivity and life stage capacity by modifying habitat specific productivities and densities at the reach scale. The five habitat quality elements that affect capacity and productivity are: barriers, fine sediment, wood, temperature and impervious area. Habitat quality attributes are calculated for each 200m reach and are represented by a 0 to 1 multiplier. Life stage and reach specific habitat quality is the product of habitat quality metrics:

$\beta_{l,r}=\prod_{s} \beta_{l,r,s}$,

where $\beta_{l,r,s}$ is the multiplier for a given life stage *l*, reach *r* and habitat quality attribute *s,* and $\beta_{l,r}$ is the final quality multiplier for a given life stage and reach. Species and life stage specific details are given in S3.

## Capacity

For each spatial unit a capacity is calculated for each density-dependent life stage. The first step is to calculate the life stage-specific capacity for each habitat type at the reach level. This is done by summing the areas of each habitat unit within a given reach. The habitat area is then multiplied by the corresponding salmon density and habitat quality multipliers.

To calculate the reach level capacity of habitat type *h,* in reach *r,* for a given life stage *l*, we sum the individual units *j,* and multiply by density *d* and the habitat quality multiplier $\beta$:

$c_{l,r,h}=\sum_{j} A_{l,r,h,j}\cdot d_{l,h}\cdot\beta_{l,r}$,

where

- *c_l,r,h_* is the capacity of life stage *l,* reach *r,* habitat type *h*
- Σ*A_l,r,h,j_* is the sum of area *A* for life stage *l,* reach *r,* habitat type *h,* habitat unit *j*
- *d_l,h_* is the density of fish in life stage *l,* and habitat type *h* (densities of fish in each habitat unit type are in Table S3-1)
- $\beta_{l,r}$ is the habitat quality multiplier for life state *l* and reach *r*

The subbasin capacity for a single life stage, $c_{l}$, is the sum of capacities for all reaches, *r*, and all habitat types, *h*:

$c_{l}=\sum_{r} \sum_{h} c_{l,r,h}$.

Because of the spatial overlap of spring and fall Chinook salmon spawning and rearing, we apportioned the estimated capacity for total estimated Chinook salmon to the two runs according to three subbasins that had estimated escapements of each run type --Newaukum River, Skookumchuck River, and South Fork Chehalis River -- and used the average 65%/35% (fall/spring Chinook salmon) from these three tributaries for subbasins where we didn’t have escapement information. In the mainstem Chehalis River subbasins we used an 81%/19% (fall Chinook/spring Chinook salmon) split rule based on overall historical Chehalis basin escapement information, assuming that fall Chinook salmon fry arrive from tributary subbasins.

## Productivity

The spatial structure of the life-cycle models requires a single value of productivity for each species, life stage, and subbasin. We use empirical or modeled productivity values (*p*) for each life stage and species representing current conditions. We calculate the weighted average productivity for each subbasin based on the proportion of capacity in each habitat type for each reach. The weighting function is:

$w_{l,r,h}= \frac{c_{l,r,h}}{c_{l}}$,

where $w_{l,r,h}$ is the weighting value for habitat type *h*, reach *r,* and life stage *l*. The weighted average productivity for a given life stage is then:

$p_{l}=\sum_{r} p_{l,h}{\cdot\beta}_{l,r}\cdot w_{l,r,h}$,

where

- $p_{l}$ is the weighted average productivity for life stage *l*
- $p_{l,h}$ is the productivity of habitat type *h* and life stage *l*
- $\beta_{l,r}$ is the habitat quality multiplier for life stage *l* and reach *r*

Values of *p_l_* for each species, life history stage, and habitat type are in Table S3-2. In density dependent life stages we use the capacity to weight the productivity. Density independent life stages are the simple average of all habitat specific productivities.

# S3. Model Parameters

The models use three main parameter types to calculate capacity and survival for each life stage: fish density, productivity, and maturation rate. Below we summarize the baseline values for each parameter type, which represent current habitat conditions and age structure of populations.

## Density

The baseline densities for each of the models are the habitat- and species-specific density values we expect under current conditions of low wood abundance and T <18°C. These baseline values are then modified by three habitat quality modifiers: passage, temperature and wood.

Table S3-1. Data used to estimate life stage capacities in the life cycle models for the Chehalis River basin. Chinook salmon includes both spring and fall runs. Data from: (1–5), and J. Thompson (City of Seattle, pers. comm.). Where second year rearing densities were unavailable for steelhead, we set density at 31% of first year densities based on Winkowski et al. (6). For steelhead spawning (number of eggs), we include first time and repeat spawners, respectively.

|  |  | **Density (per m^-2^)** | | |
| --- | --- | --- | --- | --- |
| **Rearing Life Stage** | **Habitat Type** | **Chinook** | **Coho** | **Steelhead** |
| Spawning (eggs)  First year summer rearing -- age 0+ coho and steelhead  Fry colonization – spring and fall Chinook | Pool tail/riffle crest  Large river  Bank (natural)  Bank (modified)  Bar (boulder)  Bar (gravel)  Bar (sand)  Backwater  Mid-channel  Small stream  Pool (sm. stream)  Riffle (sm. stream)  Beaver pond (<5 ha)  Floodplain  Lake (>5 ha)  Marsh  Side-channel pool  Side-channel riffle  Floodplain slough/pond (<500 m^2^)  Floodplain slough/pond (>500 m^2^) | 382  1.27  0.64  0  0.64  0.32  1.91  0.0038  0.05  0.02  0.05  0  0  0.05  0.02  0.05  0.05 | 417  1.96  0.96  0  0  0  1.86  0  1.7  0.3  1.2  0  0  1.7  0.3  1.8  0.9 | 1,000 ;1,481  1.27  0.64  1.54  1.54  0  1.27  0.064  0.70  0.53  0  0  0  0.70  0.53  0  0 |
| First year winter rearing – age 1 coho and steelhead  Sub-yearling rearing – spring and fall Chinook | Bank (natural)  Bank (modified)  Bar (boulder)  Bar (gravel)  Bar (sand)  Backwater  Mid-channel  Pool (sm. stream)  Riffle (sm. stream)  Beaver pond  Lake (>5 ha)  Marsh  Side-channel pool  Side-channel riffle  Floodplain slough/pond (<500 m^2^)  Floodplain slough/pond (>500 m^2^) | 1.27  0.64  0  0.64  0.32  1.91  0.0038  0.05  0.02  0.05  0  0  0.05  0.02  0.05  0.05 | 0.32  0  0  0  0  0.64  0  0.4  0.01  1.2  0.0025  0.32  0.4  0.01  1.8  0.9 | 0.31  0.31  0.31  0.31  0  0  0.016  0.16  0.11  0  0  0.16  0.11  0.03  0  0 |
| Second and third year summer rearing -- age 1+ and 2+ (Beverton-Holt) | Bank (natural)  Bank (modified)  Bar (boulder)  Bar (gravel)  Bar (sand)  Backwater  Mid-channel  Pool (sm. stream)  Riffle (sm. stream)  Beaver pond (<5 ha)  Lake (>5 ha)  Marsh  Side-channel pool  Side-channel riffle  Floodplain pond (<5 ha)  Slough  Marsh | NA | NA | 0.34  0.20  0.23  0.23  0  0.13  0.109  0.18  0.07  0.07  0  0  0.18  0.07  0.07  0  0 |
| Second and third year winter rearing – age 2 and 3 (Beverton-Holt) | Bank (natural)  Bank (modified)  Bar (boulder)  Bar (gravel)  Bar (sand)  Backwater  Mid-channel  Pool (sm. stream)  Riffle (sm. stream)  Beaver pond (<5 ha)  Lake (>5 ha)  Marsh  Side-channel pool  Side-channel riffle  Floodplain pond (<5 ha)  Slough  Marsh | NA | NA | 0.096  0.096  0.096  0.096  0  0.005  0.027  0.09  0.04  0.01  0  0  0.09  0.04  0.01  0  0 |
|  |  |  |  |  |

## Productivity

Productivities for current conditions (with T <18°C) are the baseline productivities for the life cycle models. They vary by species, life stage, and habitat type. Where we have productivity data, habitat-specific productivity $p_{l,h}$ is the 75th percentile of observed survivals. In all other cases they are reported productivities.

Table S3-2. Life stage productivities in the life cycle models for each species in the Chehalis River basin under current conditions. Productivities are a function of (as described in S1) and modified by habitat conditions as described in S2. Data from (7–14).

|  |  | **Productivity (current conditions)** | | |
| --- | --- | --- | --- | --- |
| **Rearing Life Stage** | **Habitat Type** | **Chinook salmon** | **Coho** | **Steelhead** |
| Prespawn |  | 1.0 | 0.95 | 0.95 |
| Spawning |  | 5,400 | 2,500 | First 5,400; Respawn 8,000 |
| Incubation |  | NA | NA | NA |
| First year summer rearing -- age 0+ coho and steelhead    Fry colonization – spring and fall Chinook | Large river  Small stream  Floodplain | 0.89  0.89  0.94 | 0.84  0.84  0.84 | 0.6  0.6  0.74 |
| First year winter rearing – age 1 coho and steelhead  Sub-yearling rearing – spring and fall Chinook | Large river  Small stream  Small stream - beaver pond  Floodplain | 0.29  0.29  0.53  0.53 | 0.35  0.35  0.78  0.78 | 0.35  0.35  0.52  0.52 |
| Second and third year summer rearing -- age 1+ and 2+ (Beverton-Holt) | Large river  Small stream  Floodplain | NA | NA | 0.85  0.85  0.74 |
| Second and third year winter rearing – age 2 and 3 (Beverton-Holt) | Large river  Small stream  Floodplain | NA | NA | 0.49  0.49  0.52 |
|  |  |  |  |  |

## Delta-bay productivity, ocean productivity, and maturation rates

For each species, smolt-to-adult return (SAR) rates represent survival of fish from delta entry at the end of freshwater rearing to river entry at the end of ocean rearing (Table S3-3). For this analysis, SAR rates for coho and steelhead were fixed but for Chinook salmon they varied slightly with the proportion of fry and subyearling outmigrants. We have literature estimates of annual ocean survival rates and local data quantifying age structure of adult returns. The one productivity value we do not have is a survival rate through the delta-bay. For this estimate, we divided SAR by the average ocean survival (weighted average across age classes and outmigrant groups) to back calculate delta-bay productivity for each species and outmigrant group. We used this procedure to estimate delta-bay productivities for fry and subyearling migrants for Chinook salmon. Maturation rates (percent of fish in each ocean age class returning to spawn) were calibrated to match the observed age structure of adult returns for each species. Only steelhead have respawners. The cumulative respawn rate for steelhead (product of the four values in Table S3-3) is 12%, which is within the range of values reported for coastal populations in the western United States (Clemens 2015).

Table S3-3. Estimated delta-bay and ocean productivities in the life cycle models for all species modeled in the Chehalis River basin. Dashes indicate that the life stage or age group is not applicable to that species. Data from (15–18). The values above the double line are the recommended SARs, and the values below are the parameters used to model that recommendation. The “*” notation indicates where the proportion of fry and subyearling migrants is dictated by density dependence.

| **Parameter** | **Age** | **Coho** | **Spr. Chinook** | **Fall Chinook** | **Steelhead** |
| --- | --- | --- | --- | --- | --- |
| Guideline SAR | --- | 0.04 | 0.004 | 0.004 | 0.08 |
| LCM-modeled SAR | --- | 0.04 | 0.0043 | 0.0019 | 0.08 |
| Outmigration rate  Delta-bay productivity | Fry/Subyr  1+  2+  3+  Fry migrant  Subyearling  Age 1  Age 2  Age 3 | 0  1.0  0  0  -  -  0.08  -  - | *  0  0  0  0.001  0.06  -  -  - | *  0  0  0  0.001  0.06  -  -  - | 0  0.003  0.7  0.297  -  -  0.14  0.14  0.14 |
| Ocean productivity | Age 1  Age 2  Age 3  Age 4  Age 5  Age 6 | -  0.7  0.7  -  -  - | 0.6  0.7  0.8  0.9  0.9  - | 0.6  0.7  0.8  0.9  0.9  - | -  0.8  0.8  0.8  0.8  0.8 |
| Maturation rate | *b*_2_  *b*_3_  *b*_4_  *b*_5_  *b*_6_ | 0.033  1  -  -  - | 0.005  0.097  0.6  0.8  1 | 0.008  0.07  0.35  0.85  1 | -  0.01  0.43  0.76  1 |
| Respawn components  Kelt rate  Reconditioning of kelts in ocean  Return rate | | NA | NA | NA | 0.4  0.6  0.5 |

# S4. Parameters Scaled with Habitat Quality

Each of these observed density or productivity values may be modified by reach specific habitat quality. The analysis of the habitat quality and changes to habitat areas are detailed in the companion paper (Beechie et al. this volume). This section describes the way changes to habitat alter density and productivity values.

## Barrier Multiplier

We use the cumulative passage percentage to directly scale prespawn productivity and spawning density. Rearing capacity is not affected by a barrier unless the passage rating is 0, at which point we assume there is zero rearing potential:

$\beta_{passage}= \prod_{b} \beta_{b}$,

where $\beta_{passage}$ is the cumulative passage multiplier for each reach and $\beta_{b}$ is the passage rating of each barrier downstream of a given reach.

## Fine sediment multiplier

Fine sediment impacts the incubation productivity. Reach specific incubation productivity comes from a reach level fine sediment estimate using the equation (10):

$\beta_{sed}=\frac{1}{{1+e}^{{-(\beta}_{0}+\beta_{1}*sed)}}$’

where $\beta_{sed}$ is incubation productivity, $sed$ is percent fine sediment < 0.85 mm, and $\beta_{0}$ and $\beta_{1}$ are empirically derived ($\beta_{0}$ = 1.989 and $\beta_{1}$ = -0.185). The final subbasin productivity is the average of reach level productivities.

## Wood multiplier

In addition to habitat area changes described in Beechie et al. (this volume), wood can positively change both productivity and density for the rearing stages of all species. For small streams, the wood multiplier applies to all habitat types evenly. While in large river reaches it impacts bar habitats and bank habitats differently. The large river wood multiplier is applied to all large river habitat units as the subbasin average weighted by length of bar and bank habitat (Table S4-1).

Table S4-1: Natural state condition wood multipliers by life stage, species, and habitat types ((14,19)).

|  |  | **Wood multiplier** | | |
| --- | --- | --- | --- | --- |
| **Rearing Life Stage** | **Habitat Type** | **Chinook** | **Coho** | **Steelhead** |
| First year summer rearing -- age 0+ coho and steelhead;  Fry colonization – spring and fall Chinook | Pool, riffle, side-channel pool,  Side-channel riffle  Edge habitat: bar  Edge habitat: bank | 1.04  1.002  1.01 | 1.07  1.02  1.20 | 1.03  1.02  1.20 |
| First year winter rearing – age 1 coho and steelhead  Sub-yearling rearing – spring and fall Chinook | Pool, riffle, side-channel pool,  Side-channel riffle  Edge habitat: bar  Edge habitat: bank | 1.59  1.03  1.15 | 1.66  1.20  1.21 | 1.66  1.20  1.21 |
| Second and third year summer rearing -- age 1+ and 2+ | Pool, riffle, side-channel pool,  Side-channel riffle  Edge habitat: bar  Edge habitat: bank | NA | NA | 1.04  1.20  1.21 |
| Second and third year winter rearing – age 2 and 3 | Pool, riffle, side-channel pool,  Side-channel riffle  Edge habitat: bar  Edge habitat: bank | NA | NA | 1.08  1.20  1.21 |
|  |  |  |  |  |

## Temperature multipliers

### Temperature effect on spring Chinook adult migration and holding productivity

For spring Chinook upstream migration and holding, we model the life stage as density independent with productivities estimated as a function of subbasin-averaged stream temperature. The equation for calculating spring Chinook prespawn productivity is based on Bowerman et al. 2018, and assumes no hatchery fish:

$\beta_{ps}=1-\frac{e^{(-9.053 + .387T)}}{1+e^{(-9.053+ .387T)}}$,

where *T* is the 7-day average of daily maximum stream temperatures.

### Temperature effect on coho salmon summer rearing capacity and productivity

Increasing stream temperature decreases coho salmon abundance and productivity via changes in summer rearing capacity and productivity (20). We used a reach specific multiplier using the form:

$\beta_{t,coho}=\left\{ \begin{aligned} 1 if T<17^{\circ}C \\ 1-0.09(T-17) if T 17^{\circ}C \leq T< 28^{\circ}C \\ 0 if T>28^{\circ}C \end{aligned} \right.$,

where *T* is the 7-day average of daily maximum stream temperatures.

### Temperature effect on steelhead summer rearing capacity and productivity

For steelhead, we employed a multiplier using an experimentally derived relationship between juvenile steelhead survival and stream temperature (21). The regression equation is:

$\beta_{t, sthd}=\frac{97.88}{1-e^{-\left( \left( T-24.3522 \right)/-0.5033 \right)}}$,

where *T* is the 7-day average of daily maximum.

### Temperature effect on Chinook rearing capacity and productivity

For spring and fall Chinook, we use the average daily maximum temperature from June 1-21 to estimate the temperature effect on subyearling rearing productivity. Based on data from (22), we estimate that with the increasing temperatures 45% of parr are affected, which is the proportion of Chinook parr counted in the first 3 weeks in June. To calculate the rearing temperature multiplier $\beta_{t, chino}$, we use the same relationship as $\beta_{t,coho}$ but where *T* is the average daily maximum temperature from June 1-21.

## Impervious surface multiplier

We calculate the percent impervious surface upstream of each reach. This reach-based impervious area is then used to estimate a prespawn productivity multiplier $\beta_{imperv}$ using the equation below (23,24):

$$\beta_{imperv}=\left\{ \begin{aligned} 0 if I>66.67\% \\ 1-0.015I if I\leq66.67\% \end{aligned} \right.,$$

where $I$ is the percent impervious surface area draining to a given reach. For historical conditions we set percent impervious area to zero.

References

1. Johnson SL, Solazzi MF, Rodgers JD. Development and Evaluation of Techniques to Rehabilitate Oregon’s Wild Salmonids. Oregon Department of Fish and Wildlife; 1993 p. 1–12.

2. Beechie T, Beamer E, Wasserman L. Estimating Coho Salmon Rearing Habitat and Smolt Production Losses in a Large River Basin, and Implications for Habitat Restoration. North American Journal of Fisheries Management. 1994;14(4):797–811.

3. Nickelson TE. A Habitat-Based Assessment of Coho Salmon Production Potential and Spawner Escapement Needs for Oregon Coastal Streams. In 1998.

4. Beamer EM, Henderson RA. Juvenile Salmonid Use of Natural and Hydromodified Stream Bank Habitat in the Mainstem Skagit River, Northwest Washington. La Conner, Washington: Skagit System Cooperative Research Department; 1998.

5. Henning J. An Evaluation of Fish and Amphibian Use of Restored and Natural Floodplain Wetlands. Washington Department of Fish and Wildlife; 2004 p. 90.

6. Winkowski J, Walther E, Zimmerman M. Summer Riverscape Patterns of Fish, Habitat, and Temperature in Sub Basins of the Chehalis River, 2013-2016. 2018;130.

7. Reeves GH, Everest FH, Nickelson TE. Identification of physical habitats limiting the production of coho salmon in western Oregon and Washington. Pacific Northwest Research Station: United States Department of Agriculture; 1989. Report No.: 245.

8. Mobrand Biometrics, Inc. Assessment of Salmon and Steelhead Perfomance in the Chehalis River Basin in Relation to Habitat Conditions and Strategic Prioritites for Conservation and Recovery Actions. Vashon, Washington: Mobrand Biometrics, Inc.; 2003.

9. Harvey BC, White JL, Nakamoto RJ. Habitat-specific biomass, survival and growth of rainbow trout (Oncorhynchus mykiss) during summer in a small coastal stream. Canadian Journal of Fisheries and Aquatic Sciences. 2005;62:650–8.

10. Jensen D, Steel E, Fullerton A, Pess G. Impact of Fine Sediment on Egg-To-Fry Survival of Pacific Salmon: A Meta- Analysis of Published Studies. Reviews in Fisheries Science. 2009 Jun 17;17:348–59.

11. Grantham TE, Newburn DA, McCarthy MA, Merenlender AM. The Role of Streamflow and Land Use in Limiting Oversummer Survival of Juvenile Steelhead in California Streams. Transactions of the American Fisheries Society. 2012 May 1;141(3):585–98.

12. Martens KD, Connolly PJ. Juvenile Anadromous Salmonid Production in Upper Columbia River Side Channels with Different Levels of Hydrological Connection. Transactions of the American Fisheries Society. 2014 May 4;143(3):757–67.

13. Ogston L, Gidora S, Foy M, Rosenfeld J. Watershed-scale effectiveness of floodplain habitat restoration for juvenile coho salmon in the Chilliwack River, British Columbia. Can J Fish Aquat Sci. 2014 Nov 13;72(4):479–90.

14. McHugh PA, Saunders WC, Bouwes N, Wall CE, Bangen S, Wheaton JM, et al. Linking models across scales to assess the viability and restoration potential of a threatened population of steelhead (Oncorhynchus mykiss) in the Middle Fork John Day River, Oregon, USA. Ecological Modelling. 2017 Jul 10;355:24–38.

15. Godfrey H. Chinook and Coho Salmon Hatchery Evaluation Studies Eighth Progress Report. Fisheries Research Board of Canada; 1969 p. 1–42. (Manuscript Report Series No. 1043). Report No.: 8.

16. Ricker WE. Review of the Rate of Growth and Mortality of Pacific Salmon in Salt Water, and Noncatch Mortality Caused by Fishing. J Fish Res Bd Can. 1976 Jul 1;33(7):1483–524.

17. Howell PJ. Stock Assessment of Columbia River Anadromous Salmonids : Final Report, Volume II, Steelhead Stock Summaries, Stock Transfer Guidelines, Information Needs. In 1985.

18. Greene, Correigh M., Beechie TJ. Habitat-specific population dynamics of ocean-type chinook salmon (Oncorhynchus tshawytscha) in Puget Sound. Canadian Journal of Fisheries & Aquatic Sciences. 2004;61:590–602.

19. Quinn TP, Peterson NP. The influence of habitat complexity and fish size on over-winter survival and growth of individually marked juvenile coho salmon (Oncorhynchus kisutch) in Big Beef Creek, Washington. Can J Fish Aquat Sci. 1996 Jul 1;53(7):1555–64.

20. Richter A, Kolmes SA. Maximum Temperature Limits for Chinook, Coho, and Chum Salmon, and Steelhead Trout in the Pacific Northwest. Reviews in Fisheries Science. 2005 Feb 23;13(1):23–49.

21. Bear EA, McMahon TE, Zale AV. Comparative Thermal Requirements of Westslope Cutthroat Trout and Rainbow Trout: Implications for Species Interactions and Development of Thermal Protection Standards. Transactions of the American Fisheries Society. 2007 Jul;136(4):1113–21.

22. Winkowski J, Zimmerman M. Chehalis River Smolt Production 2018. Olympia, Washington: Washington Department of Fish and Wildlife; 2019 p. 42. Report No.: FPA 19-01.

23. Feist BE, Buhle ER, Arnold P, Davis JW, Scholz NL. Landscape Ecotoxicology of Coho Salmon Spawner Mortality in Urban Streams. PLOS ONE. 2011 Aug 17;6(8):e23424.

24. Feist BE, Buhle ER, Baldwin DH, Spromberg JA, Damm SE, Davis JW, et al. Roads to ruin: conservation threats to a sentinel species across an urban gradient. Ecological Applications. 2017;27(8):2382–96.
